# Impact of a community health worker HIV treatment and prevention intervention in an HIV hotspot fishing community in Rakai, Uganda (mLAKE): study protocol for a randomized controlled trial

**DOI:** 10.1186/s13063-017-2243-6

**Published:** 2017-10-23

**Authors:** Larry W. Chang, Ismail Mbabali, Xiangrong Kong, Heidi Hutton, K. Rivet Amico, Caitlin E. Kennedy, Fred Nalugoda, David Serwadda, Robert C. Bollinger, Thomas C. Quinn, Steven J. Reynolds, Ronald Gray, Maria Wawer, Gertrude Nakigozi

**Affiliations:** 1 Division of Infectious Diseases, Department of Medicine, Johns Hopkins School of Medicine, Baltimore, MD USA; 2 Department of Epidemiology, Johns Hopkins Bloomberg School of Public Health, Baltimore, MD USA; 3 Department of International Health, Johns Hopkins Bloomberg School of Public Health, Baltimore, MD USA; 4 Rakai Health Sciences Program, Rakai, Uganda; 5 Department of Psychiatry and Behavioral Sciences, Johns Hopkins School of Medicine, Baltimore, MD USA; 6 Department of Health Behavior Health Education, University of Michigan, Ann Arbor, MI USA; 7 Laboratory of Immunoregulation, Division of Intramural Research, National Institute for Allergy and Infectious Diseases, National Institutes of Health, Bethesda, MD USA

**Keywords:** HIV, Antiretroviral therapy, Randomized controlled trial, Community health workers, mHealth, Uganda, Male circumcision, Motivational interviewing, Adherence

## Abstract

**Background:**

Effective yet practical strategies are needed to increase engagement in HIV treatment and prevention services, particularly in high-HIV-prevalence hotspots. We designed a community-based intervention called “Health Scouts” to promote uptake and adherence to HIV services in a highly HIV-prevalent fishing community in Rakai, Uganda. Using a situated Information, Motivation, and Behavioral skills theory framework, the intervention consists of community health workers, called Health Scouts, who use motivational interviewing strategies and mobile health tools to promote engagement in HIV treatment and prevention services.

**Methods/design:**

The Health Scout intervention is being evaluated through a pragmatic, parallel, cluster-randomized controlled trial with an allocation ratio of 1:1. The study setting is a single high-HIV-prevalence fishing community in Rakai, Uganda divided into 40 contiguous neighborhood clusters each containing about 65 households. Twenty clusters received the Health Scout Intervention; 20 clusters received standard of care. The Health Scout intervention is delivered within the community at the household level, targeting all residents aged 15 years or older. The primary programmatic outcomes are self-reported HIV care, antiretroviral therapy, and male circumcision coverage; the primary biologic outcome is population-level HIV viremia prevalence. Follow-up is planned for about 3 years.

**Discussion:**

HIV treatment and prevention service engagement remains suboptimal in HIV hotspots. New, community-based implementation approaches are needed. If found to be effective in this trial, the Health Scout intervention may be an important component of a comprehensive HIV response.

**Trial registration:**

ClinicalTrials.gov, ID: NCT02556957. Registered on 20 September 2015.

**Electronic supplementary material:**

The online version of this article (doi:10.1186/s13063-017-2243-6) contains supplementary material, which is available to authorized users.

## Background

Combination HIV prevention (CHP) is the application of multiple HIV-prevention interventions, such as antiretroviral therapy (ART), and male circumcision (MC), to maximize population-level impact on HIV incidence [1, 2]. Treatment and prevention service cascades—which depict engagement and loss of service users and people living with HIV along the continuum of service delivery—have fostered skepticism about whether high CHP coverage goals are realistic [3–5], and highlight the need for effective and sustainable implementation strategies that are not primarily facility-based [6]. Current studies to evaluate CHP mostly involve well-resourced approaches, limiting reproducibility and sustainability [7]. Empirical testing of CHP implementation approaches that are practical and community-based are, therefore, needed.

HIV hotspots are geographically defined areas of extremely high HIV transmission and prevalence. Hotspots are found throughout sub-Saharan Africa (e.g., certain mining and fishing communities), and may compromise national and regional HIV prevention efforts [8–17]. The President’s Emergency Plan for AIDS Relief (PEPFAR), the Joint United National Program on HIV/AIDS (UNAIDS), and the World Health Organization (WHO) have made HIV hotspots a central focus of their HIV response [18–20]. However, rigorous evidence on comprehensive and effective CHP strategies for hotspots is limited and, therefore, a priority research need [7]. Many Lake Victoria fishing communities, the setting for this trial, are hotspots due to a number of factors increasing HIV risk, including high rates of condomless sex, multiple sex partners, prominent sex work, and prevalent alcohol misuse [10, 21].

Fishing communities represent a critical challenge to HIV prevention in Uganda and the wider region. The fishing community hosting this research has the highest HIV prevalence, approximately 40%, ever reported from the Lake Victoria region [21]. It is a place where HIV prevention and service needs are great, yet resources and health system infrastructure are modest. A community-based approach offers an opportunity to work with the community to devise potential solutions to HIV-related challenges. Successful HIV efforts in this setting may provide a model for similar settings.

Community health workers (CHWs), using mobile health technologies (mHealth) and motivational interviewing (MI) strategies to tailor intervention messaging, may represent a high-impact and sustainable CHP implementation strategy, particularly in HIV hotspots [22–26]. CHW impact could be optimized by mHealth tools as sub-Saharan Africa has some of the fastest mobile phone growth rates in the world [27, 28]. CHW effectiveness could be further enhanced using evidence-based, MI counseling techniques that emphasize a client-centered approach. We therefore designed the “Health Scouts” mLAKE trial (mHealth Lakefolk Actively Keeping Engaged) which is a CHW-based, mHealth-supported, MI-informed, theory-based CHP implementation intervention being evaluated in an HIV hotspot fishing community in Rakai, Uganda. MI was chosen as the foundation of the CHW counseling strategy because of its cross-cultural robustness, and effectiveness with heterogeneous populations [29, 30]. HIV hotspots may particularly benefit from the MI techniques which emphasize taking into consideration complex contextual factors influencing client behaviors. mHealth could be of particular benefit in this setting in assisting CHWs through complicated counseling algorithms for individuals with multiple areas of HIV risk.

Here, we present the study protocol for the mLAKE trial which began recruitment in September 2015 with follow-up planned until at least 2018. The objective of the study is to determine the impact of the CHW intervention on important HIV-related treatment and prevention outcomes, i.e,. HIV service uptake and population prevalence of viremia. This study is being conducted in a hotspot fishing community because of the need for more effective interventions to improve treatment and prevention in high-HIV-burden, high-HIV-risk settings. The protocol format is guided by the Standard Protocol Items: Recommendations for Interventional Trials (SPIRIT) Checklist (Additional file 1) and the Consolidated Standards of Reporting Trials (CONSORT) extension to cluster-randomized trials [31, 32].

## Methods/design

### Study setting

The intervention setting is a fishing community (area approximately 2 km^2^, adolescent/adult population approximately 4400) on Lake Victoria in Rakai District in south-central Uganda. Rakai District is bordered to the south by Tanzania and to the east by Lake Victoria. The fishing community is approximately 235 km from Kampala, the capital of Uganda. Since 2011, the Rakai Health Sciences Program (RHSP) has been the primary provider of CHP services in this fishing community. These services include community-based demand generation (e.g., mass media such as radio-talk shows, community meetings, and drama shows), an HIV clinic which provides free pre-ART and ART care, community-based HIV testing, and mobile MC camps. In this community, ART is initiated at time of diagnosis, irrespective of CD4 count (i.e., test and start strategy) as fisherfolk have been designated a key population by the Ugandan Ministry of Health. The HIV prevalence in this community is approximately 40%.

### Trial design overview

**Fig. 1:**
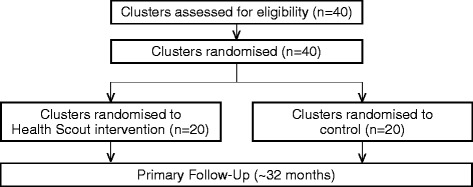
The mHealth Lakefolk Actively Keeping Engaged (mLAKE) study flow diagram

The impact of the Health Scout intervention will be assessed through a pragmatic, parallel, cluster-randomized controlled trial with an allocation ratio of 1:1 and superiority framework (Fig. 1). The cluster-randomized design was chosen over other study designs as individual and household randomized designs were logistically difficult to implement and increased contamination risks; quasi-experimental designs were deemed less rigorous.

#### Cluster design

The largest of the fishing communities in the region was selected for the mLAKE trial and 40 was chosen as the number of randomization units. There were several reasons for choosing 40 units or clusters. First, we anticipated 10 Health Scouts being deployed, each of whom would initially be allocated the same number of clusters. Some variation in the type of clusters Health Scouts were assigned was desirable to enable more robust analytic inferences on Health Scout effects. Thus, each Health Scout needed to have at least two assigned clusters. As progressively larger number of clusters increased implementation complexity and risk of contamination, we selected 40 clusters, the minimum number needed to assign each Health Scout two clusters.

**Fig. 2:**
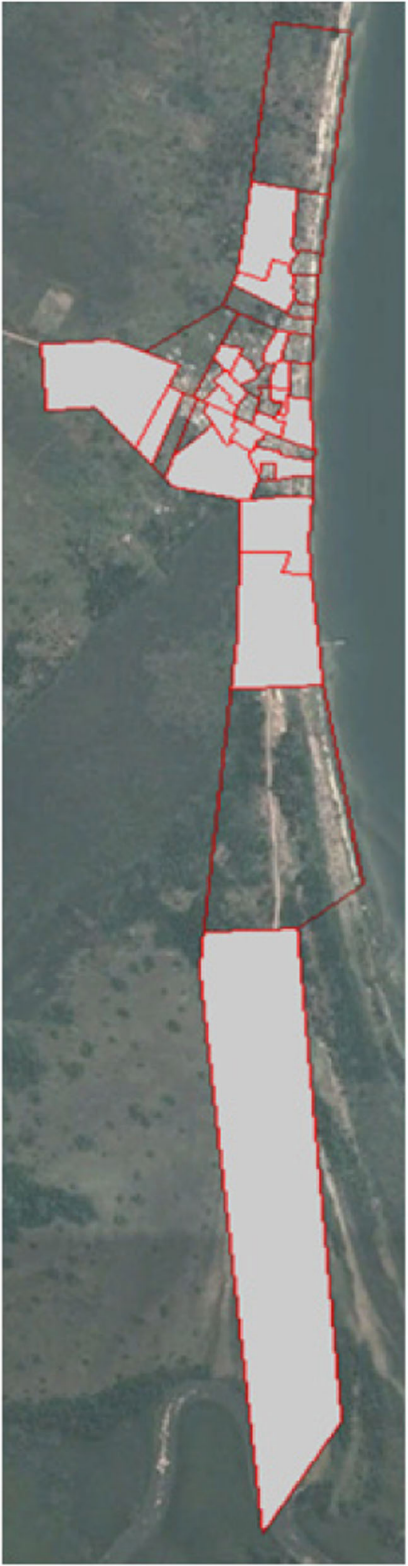
mHealth Lakefolk Actively Keeping Engaged (mLAKE) trial cluster map (intervention: clear, control; grayed)

The 40 clusters were then defined with consideration of implementation logistics, minimizing contamination, and geographic features such as roads, buildings, and the lake shoreline. Specifically, high-resolution satellite images of the community were digitized in ArcGIS 10.3 (ESRI, Redlands, CA, USA). Household GPS locations from a baseline community census conducted in mid-2015 were overlaid on the digitized map and the community was divided into 40 contiguous clusters (Fig. 2) each containing roughly the same number of eligible households and participants. Each cluster was estimated, using census data, to contain approximately 65 households with approximately 1.65 persons aged over 15 years (target population) in each household or approximately 107 potential eligible clients per cluster. Community-based participatory walks with the Health Scouts were conducted to confirm and refine cluster boundaries prior to participant enrollment [33].

### Pragmatic design considerations

**Fig. 3:**
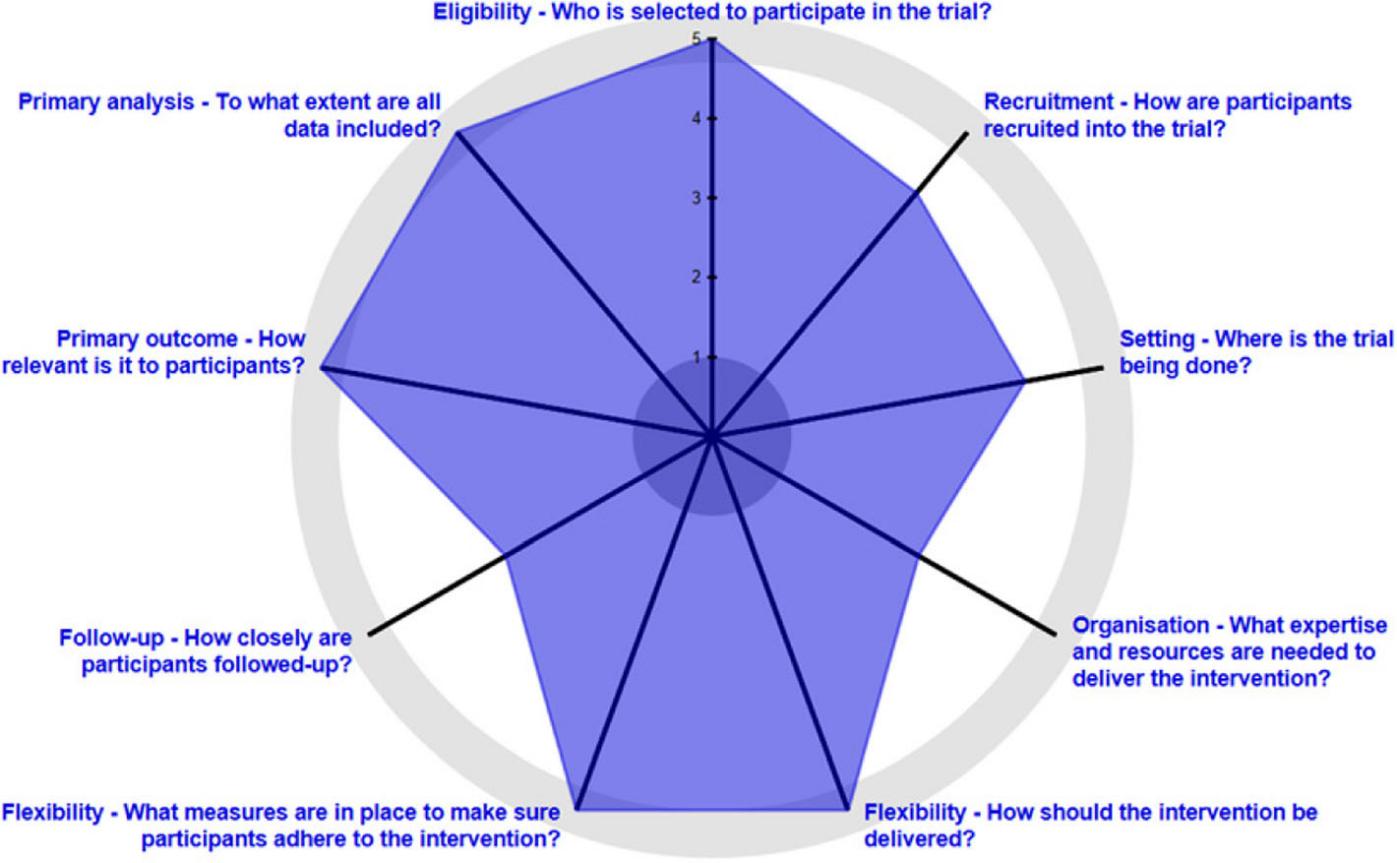
PRECIS-2 wheel for the mHealth Lakefolk Actively Keeping Engaged (mLAKE) trial

The study design is pragmatically oriented as our primary goal is to understand if the Health Scout intervention works under usual-care conditions. To explicitly describe the pragmatic characteristics of the trial, we used the PRECIS-2 tool (Table 1). This tool is a method for scoring trials on an explanatory to pragmatic continuum across nine domains. Figure 3 shows a visualization of these scores via the PRECIS-2 wheel [34].

**Table 1:** PRECIS-2 scores for the mHealth Lakefolk Actively Keeping Engaged (mLAKE) trial

Domains	Score^a^	Rationale
Eligibility	5	All residents ≥ 15 years of age are eligible for study participation, identical to usual care
Recruitment	4	Recruitment effort (community sensitization, drama shows, and Health Scouts approaching residents) is likely modestly above what would be expected in usual care
Setting	4	The setting is mostly similar in key characteristics (e.g., high-risk population) to other usual-care settings
Organization	3	The care delivery organization is likely much higher resourced than most usual-care organizations but efforts were made to replicate more usual-care resource capacity
Flexibility (delivery)	5	Flexibility is anticipated to be the same in how the intervention is delivered in the trial and flexibility anticipated in usual care
Flexibility (adherence)	5	No special trial measures to promote participant participation with Health Scout intervention
Follow-up	3	Participants being surveyed every at regular intervals in this trial which may be different from other settings
Primary outcome	5	Outcome is very directly relevant to participants
Primary analyses	5	All data will be included in the analysis of the primary outcomes

^a^PRECIS 5-point Likert Scale, 1 = very explanatory, 2 = rather explanatory, 3 = equally pragmatic/explanatory, 4 = rather pragmatic, 5 = very pragmatic

### Study hypotheses

The study hypotheses are that residents in clusters receiving the Health Scout intervention will have improved HIV service uptake (HIV care, ART, and MC) and decreased HIV population viremia prevalence (PVP) compared to residents in control clusters who do not receive the intervention.

### Health Scout intervention

#### Conceptualization

**Fig. 4:**
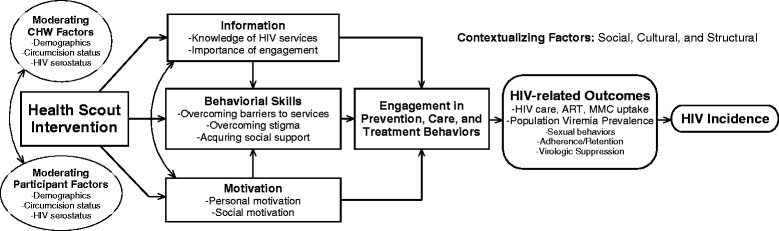
Health Scout intervention conceptual framework

The general approach to the design and implementation of the Health Scout intervention is *pragmatically* oriented [34]. That is, a framework for Health Scout recruitment, training, tasks, quality assurance, and monitoring was developed, but the intervention is designed with flexibility to adapt to the needs, constraints, and secular changes during implementation. The framework included the following core components: (1) CHW (Health Scout)-based delivery; (2) counseling using MI strategies; (3) household-based delivery; and (4) mHealth-support. A situated Information, Motivation, and Behavioral Skills (sIMB) theory-based conceptual framework was developed wherein Health Scouts would promote relevant tailored information, motivation, and behavioral skills to improve clients’ engagement in HIV treatment and prevention services, ultimately leading to improved HIV-related outcomes, including decreased HIV incidence (Fig. 4) [35, 36].

#### Implementation

Ten Health Scouts were recruited from the community and underwent residential- and field-based training. Residential training focused on mHealth application/smartphone use, confidentiality, disclosure, and CHP- and HIV-related knowledge. Field-based training focused on mock counseling role play, cluster boundary walk-throughs, and filling out Home Visit Forms. MI skills, a communication strategy that emphasizes a non-judgmental and non-confrontational approach to behavior change, were integrated into Health Scout training. After demonstrating basic competency with their assigned tasks, Health Scouts attempted to visit all “clients” (i.e., residents aged 15 years and older, any gender) within their clusters within 3 months of study initiation and follow-up each eligible client within their cluster every 3 months for approximately 3 years. During each visit, Health Scouts counsel clients with assistance from a smartphone application. Visits are anticipated to take approximately 30–45 min. Each Health Scout was assigned two clusters containing approximately 215 total eligible clients. Health Scouts were provided compensation for their activities.

#### Health Scout smartphone application

**Fig. 5:**
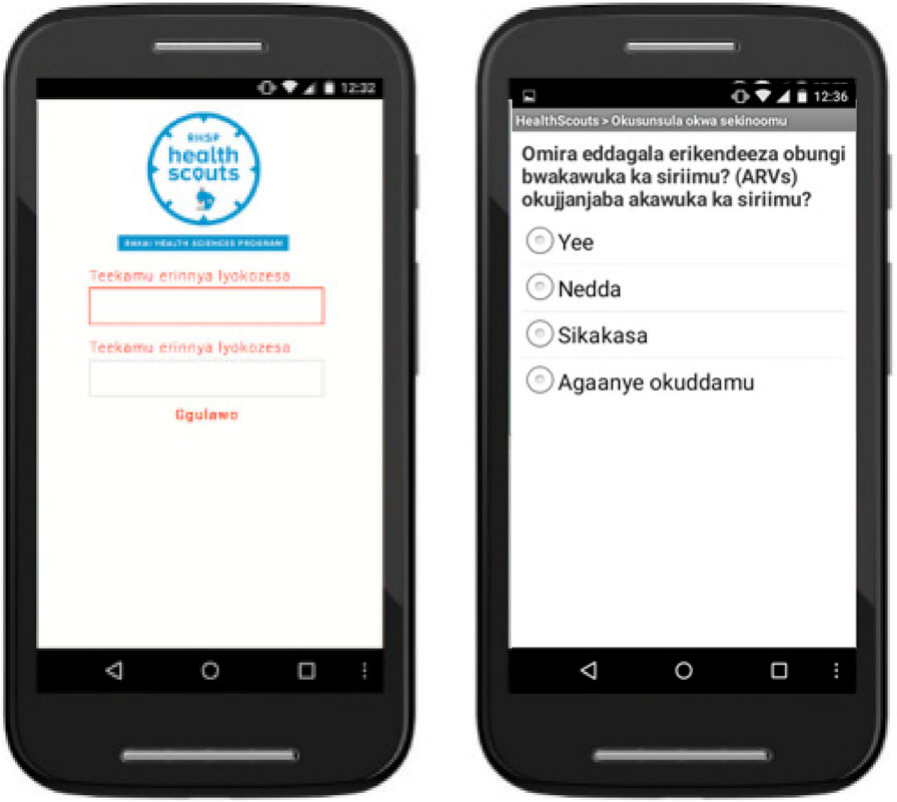
Example screenshots from Health Scout smartphone application

The Health Scout smartphone application (Fig. 5) functions as a decision and counseling support tool to guide Health Scouts through the sIMB- informed counseling session (emocha Mobile Health Inc., Baltimore, MD, USA). Using simple forms, the application takes the Health Scout through three steps: (1) household screening-household members are screened for age eligibility; (2) individualized counseling triage – residents who are age-eligible are then individually asked a series of triage screening questions, e.g., age, gender, HIV status, to determine which counseling modules should be activated; and, (3) MI counseling-tailored counseling modules with MI-informed prompts and messages are provided for the Health Scout to review with the client. The application contains nine counseling modules as shown in Table 2 (Additional file 2). Figure 6 provides a sample schematic of the counseling module content for a client who knows that they are HIV-positive, but is not yet in care.

**Table 2:** Health Scout mHealth application counseling modules

#	Module description
1	HIV serostatus unknown or no recent HIV test, male or not pregnant female
2	HIV serostatus unknown or no recent HIV test, pregnant female
3	Male, not circumcised
4	HIV+, not in care
5	HIV+, in care, not on antiretroviral therapy (ART)
6	HIV+, on ART
7	HIV+, pregnant
8	Male having condomless sex
9	Female having condomless sex

**Fig. 6:**
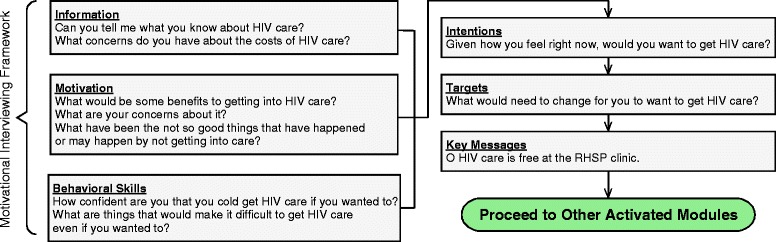
Example of counseling module flow and content: HIV-positive, not yet in care

### Control arm description

Household residents in both study arms will have access to standard of care RHSP HIV prevention and treatment services as described in the “Study setting” section above. Health Scouts are not prohibited from interacting with control arm residents but cannot conduct mHealth-supported counseling home visits with residents outside of the intervention clusters.

### Outcomes

The mLAKE trial has three primary programmatic outcomes (HIV care, ART, and MC coverage), one primary biologic outcome (HIV population viremia prevalence (PVP)), and several secondary outcomes (Table 3). PVP, variously termed community viral load, population viral load, or population prevalence of detectable viremia, was chosen as the primary biologic outcome because it is strongly correlated with HIV incidence [37]. The programmatic outcomes are all key indicators for major HIV service programs and funders.

**Table 3:** Descriptions of mHealth Lakefolk Actively Keeping Engaged (mLAKE) trial primary (1°) and secondary (2°) study outcomes^a^

	Outcome	Description	Numerator/event	Denominator
1°	HIV care coverage^b^	Proportion	# linked to HIV care	# HIV-positive
1°	ART coverage^b^	Proportion	# on antiretroviral therapy (ART)	# HIV-positive
1°	MC coverage^b^	Proportion	# non-Muslim men circumcised	All non-Muslim men aged 15–49 years
1°	HIV population viremia prevalence (PVP)	Proportion	HIV+ c VL > 400	All study participants
2°	HIV incidence	Rate	# new HIV infections	HIV-negative person-years
2°	HTS coverage	Proportion	# ever tested and received HIV results	All study participants
2°	ART treatment failure^c^	Proportion	Composite: virologic failure *or* Mortality *or* lost to follow-up *or* stopped ART	# initiated on ART
2°	Consistent condom use^b^	Proportion	# using condoms consistently past 12 months	# sexually active

^a^Outcomes described at the cluster level; outcomes may also be analyzed at the individual level

^b^Self-reported

^c^Outcomes will also be individually analyzed. Virologic failure = any viral load (VL) > 1000 copies/mL. Lost to follow-up = no clinic visit > 180 days

*ART* antiretroviral therapy, *HTS* HIV Testing Services, *MC* male circumcision

### Outcome assessment/data collection

#### Rakai Community Cohort Study (RCCS)

The mLAKE trial is nested within the RCCS which is conducted by the RHSP. The RCCS is an open, population-based cohort established in 1994 [38]. The RCCS currently surveys individuals aged 15–49 years in 40 agrarian, trading, and fishing communities in and near Rakai District, including the community where the mLAKE trial is being implemented. For all communities, the RCCS conducts a census of households with GPS coordinates recorded and all resident household members enumerated by gender, age, and duration of residence, regardless of whether they are present or absent. After census, the RCCS conducts interviews with consenting participants to assess demographics, sexual behaviors, and HIV service uptake. Data are directly entered using ultra-mobile PCs with pre-coded files and edited in the field for range and consistency checks to allow onsite correction of errors. Blood samples are collected for HIV-related tests and HIV viral load assays of HIV-positive persons. Following the interview, free HIV testing services using a three-rapid-test algorithm and post-test counseling are offered to consenting participants. The RCCS currently conducts its survey of the trial community approximately every 18 months. The existing RCCS research infrastructure allows for efficient assessment of all mLAKE trial outcomes.

#### Consent (adults)

As this is an additive counseling service being offered only to community residents randomized to the intervention arm, consent to receive the counseling service will be obtained orally by the Health Scouts. An oral consent script will be displayed on the mobile phones. Individuals will be informed that declining to receive the intervention will in no way impact their ability to receive usual care and services offered through RHSP. Health Scouts are trained to request consent according to best research practices in a confidential, non-coercive manner.

Participants in the parent RCCS study will follow the established written informed consent procedure for RCCS. The RCCS consent notes potential participation in nested studies, such as this one, allows for collection of blood samples, and allows for linking of RCCS data with available data collected originally for clinical purposes.

#### Assent/consent for minors (ages 15–17 years)

Individuals aged 15–17 years will be asked to provide oral informed assent, and their parents or guardians requested to provide concurrent oral consent. For minors who are emancipated (e.g., married or living independently), we will request oral consent from the minor only per Ugandan guidelines. If assent and consent is obtained as described above, adolescents will be interviewed and counseled in private, without the parent/guardian being present.

#### Data monitoring

Primary responsibility for data monitoring will be assumed by a Data Monitoring Committee (DMC) which will consist of study team members (senior investigators and primary analyst) without any competing interests. The DMC will oversee all data sharing, data integrity, data security, and reporting of analyses of study results. The DMC will have access to the original study database and will guide blinded primary data analyses. Study team members with potential competing interests will not be members of the DMC and will not have access to the original study database which will be kept on password-protected servers in Uganda. Access to any data by study arm and primary study outcomes will not be released until the data and related analyses have been reviewed and approved by the DMC.

#### Access to data

Only DMC members will have access to the original study database which will be kept on a password-protected server in Uganda. Data from this database will be accessible to other investigators upon request through the DMC. After trial completion and publication of primary findings, any data sharing will adhere to established data-sharing policies of RHSP.

### Randomization

#### Eligibility criteria for clusters and participants

All 40 clusters are eligible for randomization (Fig. 1). All cluster residents aged 15 years and older are eligible for participation.

#### Sequence generation, allocation concealment mechanism, and implementation

Given the fixed sample size and desire for baseline comparability of important characteristics, we used restricted randomization to allocate intervention arms [39]. First, we defined a priori that we would accept up to 7.5% difference in baseline cluster level characteristics (including percentage of residents aged 20–29 years in the cluster, percentage of women) and in primary study outcomes (including cluster level percentage of men circumcised, percentage of HIV-positive persons in care, percentage of HIV-positive persons taking ART, percentage HIV PVP). The 7.5% threshold was chosen for simplicity, consistency, and face validity. Second, we conducted 2500 independent, simple, computer-generated randomizations, assigning 20 clusters to one arm (coded as 0) and 20 clusters to a second arm (coded as 1). Third, among the 2500 randomizations, those who did not satisfy the above comparability criteria were removed, resulting in 455 eligible randomizations. Fourth, from the 455 randomizations, one randomization sequence was randomly drawn for allocation of intervention. Final assignment of arms (0 and 1) to intervention and control was done in a public coin-flip ceremony witnessed by study staff and the Health Scouts.

#### Blinding

By nature of the intervention, Health Scouts and participants will not be blinded to intervention assignment. The RCCS study team members assessing outcomes, including the primary analyst, will be blinded to intervention assignment.

### Sample size/power

The sample size for this trial is fixed. Therefore, the relevant study power-related question was to estimate whether the trial had reasonable chance to detect public health-relevant differences in primary study outcomes, i.e., minimum detectable differences (MDD). Baseline data from a mid-2015 RCCS survey of the fishing community were used to first estimate the coefficients of variation (CV) or intracluster correlation coefficients (ICC) (Table 4). We then used prior data from 9–10/2013 (approximately 18 months prior to the mid-2015 data) to project the expected prevalence of the primary outcomes expected for the control arm at the end of the trial (Table 4). The mLAKE trial is planned for approximately 36 months of follow-up, i.e., two RCCS rounds after the baseline 2015 round. The assumption is that 2013 through 2015 were times of rapid scale-up of CHP service access and that this momentum will slow (roughly by 50% for MC, HIV care and by 75% for PVP, ART, and HIV incidence) over the next 36 months.

**Table 4:** Power calculation parameters and derivation of control arm outcome estimates at the end of the trial

Characteristics	CV	ICC	2013	2015	2013 to 2015 ∆	Proposed control arm outcome
Population viremia prevalence (PVP)	0.33	0.02	25%	16%	9%	11.2%
HIV care coverage	0.141	0.061	70%	75%	4.8%	80.1%
ART coverage	0.187	0.072	43%	67%	24.6%	79.7%
MC coverage	0.209	0.062	52%	59%	8.1%	66.9%
HIV incidence	0.929	NA	3.34	2.9	0.44	2.7

*ART* antiretroviral therapy, *MC* male circumcision, *NA* not applicable

MDDs were subsequently calculated with 80% power as described by Hayes and Moulton (Table 5) [39]. Potential impact of contamination was also assessed by assuming 10–50% of control arm residents would be influenced by the intervention. Based upon these calculations, the study should have MDD ratios with 80% power to be able to detect a 41% or greater decrease in PVP, 15% or greater increase in HIV care coverage, 20% or greater increase in ART coverage, and 23% or greater increase in MC coverage. Study power was considered reasonable by the study team for all primary outcomes.

**Table 5:** Calculation of minimum detectable difference (MDD) for the mHealth Lakefolk Actively Keeping Engaged (mLAKE) trial

Outcome	Control proportion	% Contamination	Control proportion + contamination	Intervention proportion	MDD ratio	MDD difference
PVP	0.112	0	0.112	0.066	0.593	−0.046
PVP	0.112	0.1	0.107	0.063	0.559	−0.049
PVP	0.112	0.2	0.101	0.059	0.524	−0.053
PVP	0.112	0.3	0.095	0.054	0.482	−0.058
PVP	0.112	0.4	0.087	0.048	0.433	−0.064
PVP	0.112	0.5	0.077	0.041	0.37	−0.071
HIV care coverage	0.801	0	0.801	0.92	1.149	0.119
HIV care coverage	0.801	0.1	0.814	0.934	1.147	0.12
HIV care coverage	0.801	0.2	0.831	0.952	1.145	0.121
HIV care coverage	0.801	0.3	0.854	0.976	1.144	0.122
HIV care coverage	0.801	0.4	NA	NA	NA	NA
HIV care coverage	0.801	0.5	NA	NA	NA	NA
ART coverage	0.797	0	0.797	0.952	1.195	0.155
ART coverage	0.797	0.1	0.814	0.971	1.193	0.157
ART coverage	0.797	0.2	0.837	0.997	1.191	0.16
ART coverage	0.797	0.3	NA	NA	NA	NA
ART coverage	0.797	0.4	NA	NA	NA	NA
ART coverage	0.797	0.5	NA	NA	NA	NA
MC coverage	0.669	0	0.669	0.822	1.229	0.153
MC coverage	0.669	0.1	0.686	0.842	1.227	0.156
MC coverage	0.669	0.2	0.709	0.869	1.225	0.16
MC coverage	0.669	0.3	0.739	0.904	1.222	0.164
MC coverage	0.669	0.4	0.783	0.955	1.219	0.172
MC coverage	0.669	0.5	NA	NA	NA	NA
HIV incidence	0.027	0	0.027	0.005	0.201	−0.021
HIV incidence	0.027	0.1	0.025	0.005	0.176	−0.022
HIV incidence	0.027	0.2	0.022	0.004	0.146	−0.023
HIV incidence	0.027	0.3	0.02	0.003	0.116	−0.024
HIV incidence	0.027	0.4	0.017	0.002	0.08	−0.025
HIV incidence	0.027	0.5	0.014	0.001	0.05	−0.025

*ART* antiretroviral therapy*, MC* male circumcision, *NA* not applicable*, PVP* population viremia prevalence

### Participant timeline

**Fig. 7:**
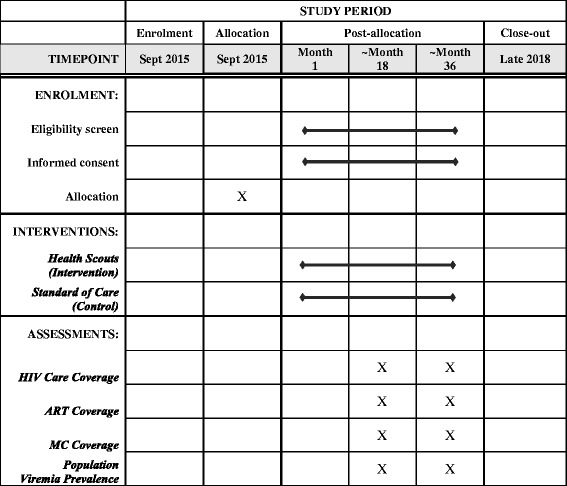
SPIRIT Figure; the mHealth Lakefolk Actively Keeping Engaged (mLAKE) trial schedule of enrollment, interventions, and assessments

Participants were recruited beginning September 2015. As shown in the SPIRIT Figure (Fig. 7), participants will be recruited in an ongoing fashion and followed for two rounds of RCCS or approximately 36 months (usually approximately 18 months between rounds). In-migrating participants can be recruited at any time they are encountered by Health Scouts in an intervention cluster during the study period. Participants can also out-migrate or decline participation at any point in time. Process and qualitative evaluations will be conducted during and after the follow-up period. A cost analysis is planned after trial completion.

### Participant recruitment

mLAKE trial-specific recruitment began with a community-wide sensitization in which community leaders along with RHSP staff informed the community about the mLAKE trial. They explained the randomized nature of the study using locally appropriate examples. This process had been successfully implemented in previous randomized trials and resulted in excellent community support for RHSP research [40]. When the Health Scouts were subsequently deployed, they began recruiting community residents to receive the counseling service as described above. Recruitment for the RCCS will follow usual procedures [21].

### Statistical methods

The comparability of intervention and control arms will be assessed at baseline. Two-sample *t* tests will be used to compare the baseline cluster-level outcomes (individual-level data at baseline are not available for participants absent from the baseline survey). Additionally, participant demographics (age, sex) are known risk factors for the study outcomes, and will be assessed for comparability. If a baseline outcome or demographic variable are significantly different between arms with *p* value ≤ 0.1, adjusted regression analysis on the outcome variables will be conducted adjusting for baseline covariates.

The cluster-level data for study outcomes will be presented by survey and by arm. The primary analysis will be by intent-to-treat and use generalized linear models (GLM) with generalized estimating equation (GEE) to account for within-cluster correlations. Specifically, the logistic model with GEE will be used for binary outcomes (e.g., PVP, MC, ART), and Poisson log-linear model with GEE will be used for the HIV-incidence outcome. Exchangeable correlation structure will be used in the GEE models. Separate analysis will be conducted for each RCCS follow-up survey to assess the shorter- and longer-term effect of the intervention.

### Study monitoring

We will continuously monitor study implementation by analyzing process data. These data may be used to adapt/reinforce the pragmatic Health Scout intervention, e.g., retraining on certain aspects of MI. All modifications will be documented in detail. A questionnaire module will be developed and affixed to the RCCS survey to assess participants’ knowledge, attitudes, and interaction with the intervention. These data will help determine intervention fidelity and reach, and will allow assessment of the magnitude of any contamination.

#### Harms

Potential harms and adverse events will be routinely monitored and reported by study staff and the principal investigator according to NIH guidelines. Staff will be trained to complete an Adverse Event Form or a Protocol Violation Form that will be filed and electronically sent to the principal investigator for review. The principal investigator will ensure that study staff are appropriately trained on adverse event reporting. Severe adverse events (e.g., breach of confidential information) will be reported promptly to the appropriate Institutional Review Boards (IRBs).

#### Auditing

The study principal investigator (LC) will have primary responsibility for monitoring study conduct and participant safety. LC will make about biannual audit visits to the study site and conduct reviews of study materials (e.g., completion of Consent Forms, log books, Adverse Event Forms) and procedures.

### Confidentiality

Confidentially will be promoted by focused training on this topic with all Health Scouts and study staff. Health Scouts will be trained to make all possible efforts to ensure privacy during Health Scout and participant interactions. Participants may also elect to be contacted on their phone rather than to meet in person if they feel more comfortable with this mode of interaction or to meet in a place outside their home for follow-up visits.

Some study data will be collected from mobile phones, including GPS location data of residences. No names will be collected on the phones. All phones used to collect these data will be password protected and all data stored on the phones will be encrypted. Phones will be wiped clean of data after final data transfer. Data collected on the phones will be transferred to a secure server using the local cellular network or Wi-Fi. All data transfers will be done in a secure fashion using state-of-the-art encryption techniques. Data stored on the server will also be encrypted, password protected, and only made available to the relevant study team members. All geospatial data will be protected to avoid identifying participant information. In all visual presentations of this type of data, specific locations will be “blurred” or presented in an aggregate format so that individual-level identification of characteristics will not be possible. If a phone is lost or stolen, data will remain secure on the device as it will be encrypted, password protected, and can be remotely wiped.

### Protocol amendments

Important protocol modifications (e.g., changes to eligibility criteria, outcomes, etc.) will be reported to all IRBs and funders according to their usual procedures. Trial registries will also be notified and updated as appropriate. Major protocol changes will be reported in any subsequent publications.

### Ancillary and post-trial care

If the intervention is found to be effective and affordable, we will attempt to secure resources to provide the intervention for all study participants.

### Dissemination policy

Study protocol and results will be presented at scientific conferences and in peer-reviewed publications. The roles of authors will be disclosed. Authorship eligibility follows the common standards of author responsibility, conflict of interest, transparency and the recommendations of the International Committee of Medical Journal Editors.

## Discussion

HIV treatment and prevention service engagement remains suboptimal, particularly in HIV hotspots in sub-Saharan Africa. New, community-based implementation approaches which are evidence-based are needed. The mLAKE trial seeks to assess the impact of the Health Scout intervention in a pragmatic trial as one strategy for improving CHP implementation.

The Health Scout intervention has several notable features. The Health Scout intervention is theory-based, using the sIMB model for care engagement, providing grounding for its application and evaluation [35]. It incorporates a motivational interviewing approach which has only been used in a small number of programs in sub-Saharan Africa despite evidence on its effectiveness in other settings [30, 41–43]. The intervention also incorporates a novel mHealth application which acts as a counseling and decision support tool. The application is intentionally built to be user-friendly and scalable in similar resource-limited settings.

The design of this trial has some noteworthy characteristics. The cluster design is unusual as typically clusters are more geographically separate [39]. However, the chosen cluster design was a pragmatic decision to allow a reasonable design for obtaining rigorous data on intervention impact. Close monitoring and reporting of intervention reach and control arm contamination will be critical to assessing the validity of study results. Additionally, the nature of the Health Scout intervention is complex and pragmatic. Secular and deliberate changes to the intervention, participants, and setting may occur over the course of the follow-up period. Thus, the planned process and mixed-methods evaluations will be helpful in providing a more comprehensive assessment of the implementation and impact of the intervention [44].

This study has a number of potential limitations. Contamination of control clusters may decrease study power to detect any true intervention effects. Secular changes, such as greater than anticipated scale-up of CHP services in control arms, may also decrease study power. Finally, findings from this study may not be easily generalizable outside of this particular HIV hotspot setting.

In summary, the mLAKE trial seeks to test the impact of the Health Scout intervention on CHP implementation. If found to be effective in this cluster-randomized trial, the Health Scout intervention may be an important strategy as part of a comprehensive and effective HIV response.

## Trial status

The trial started recruitment in September 2015. Recruitment is ongoing and is expected to be completed by December 2018.

## Additional files

Additional file 1: SPIRIT 2013 Checklist: recommended items to address in a clinical trial protocol and related documents*. (DOC 119 kb)

Additional file 2: Rakai Health Sciences Program mLAKE (mHealth Lakefolk Actively Keeping Engaged) Application. (DOCX 92 kb)

## Abbreviations

AIDS: Acquired immunodeficiency syndrome
ART: Antiretroviral therapy
CHP: Combination HIV prevention
CHW: Community health worker
CV: Coefficient of variation
DMC: Data Monitoring Committee
HIV: Human immunodeficiency virus
ICC: Intracluster correlation coefficient
MC: Male circumcision
mLAKE: mHealth Lakefolk Actively Keeping Engaged
PVP: Population viremia prevalence
RCCS: Rakai Community Cohort Study
RHSP: Rakai Health Sciences Program
