# Supplementary material for: Impact of a community health worker HIV treatment and prevention intervention in an HIV hotspot fishing community in Rakai, Uganda (mLAKE): study protocol for a randomized controlled trial
Source: Trials. 2017 Oct 23;18:494. doi: 10.1186/s13063-017-2243-6 (PMC5654192; doi:10.1186/s13063-017-2243-6)
Supplement: Supplementary file 2 — Rakai Health Sciences Program mLAKE (mHealth Lakefolk Actively Keeping Engaged) Application. (DOCX 92 kb) [file 13063_2017_2243_MOESM2_ESM.docx]

Rakai Health Sciences Program **mLAKE (mHealth Lakefolk Actively Keeping Engaged)** Application

**MASTER FORM**

|  | | **Programming Notes:**  **-The language used should be Luganda. English provided for reference only.**  **-Within each section, a response should be required before moving to the next screen unless otherwise noted.**  **-🡪🡪🡪🡪🡪🡪🡪🡪🡪🡪 means just move on to the next screen.**  **-‘O’ = Checkbox**  **-All “[I], [B], etc.” text do NOT need to be programmed in. They are for reference only.** | | | | | | |
| --- | --- | --- | --- | --- | --- | --- | --- | --- |
| **#** | **Item** | | **Item (Luganda)** | **Coding** | **Coding (Luganda)** | **Response** | **Response (Luganda)** | **Variable**  **Name** |
|  | | **Initial Household Screen**-to discern how many are eligible for counseling | | | | | | |
| 1.0 | Empty | | Empty | Empty |  | Empty |  | NA |
| 1.1 | Record counseling attempt location. | | Wandiika endagiriro yekifo wogezezzaako okugoberera omuntu owokubudabuda | [Button (GPS)] | Kozesa GPS | 🡪🡪🡪🡪🡪🡪🡪🡪🡪🡪 |  | GPS |
| 1.2 | O New location?  O Follow-up location? | | O Endagiriro eno mpya?  O Endagiriro simpya (ekifo kino wakigezaako dda? | [Checkbox] | Teeka akayini **(√)** mu ka box | 🡪🡪🡪🡪🡪🡪🡪🡪🡪🡪 |  | NEW |
| 1.3 | Anyone present? | | Osanzeewo omuntu yenna? | Yes…………………. 1  No………………….. 0 | Yee……………….1  Nedda……………0 | If 1, skip to 1.5  If 0, proceed to 1.4 | Bwekiba 1, buuka ogende ku namba 1.5  Bweba 0, genda ku 1.4 | PRESENT |
| 1.4 | This attempt to locate is complete. | | Okugezaako kw’omulundi guno kukomye wano | [Button (Save form and close application.)] | Tereka byowandiise, olyooke oggalewo | If button pressed, save form and close application. | Nyiga eppeesa,kuuma/tereka byowandiise, oggalewo,okome awo | END1 |
| 1.5 | Greet person “My name is ___ and I am a xxxx”. | | Lamusa/buuza ku muntu oba abantu b’omumaka,.  Nkulamusizza nyabo/ssebo. Amannya gange nze______ | [Checkbox] | Teeka akayini **(√)** mu ka box | 🡪🡪🡪🡪🡪🡪🡪🡪🡪🡪 |  | GREET |
| 1.6 | Confirm that there are persons age 15 or older present for possible counseling. | | Kakasa nti waliwo omuntu oba abantu aweza/abaweza emyaka 15 oba okusingawo, abaliwo okubudabudibwa | Yes…………………. 1  No/Not sure………….. 0 | Yee…………………...1  Nedda/sekakasa……0 | If 1, skip to 1.8  If 0, proceed to 1.7 | Bwekiba 1, buuka ogende ku namba 1.8  Bweba 0, genda ku 1.7 | FIFTEEN |
| 1.7 | Thank person(s) for their time and let them know your services are only for those age 15 or older. | | Webaze omuntu/abantu abo olwokukuwa obudde bwaabwe, obategeeze nti obuyambi buno buweebwa bantu abaweza emyaka 15 nokudda waggulu | [Button (Save form and close application.)] | Tereka byowandiise, olyooke oggalewo | If button pressed, save form and close application. | Nyiga eppeesa,kuuma/tereka byowandiise, oggalewo,okom’awo | END2 |

| **Consent Process** | | | | | | | | | | | |
| --- | --- | --- | --- | --- | --- | --- | --- | --- | --- | --- | --- |
| 1.8 | | Begin counseling session with resident(s), one at a time, in private. If there are adolescents present (ages 15 to 17), start with the adults first. | Tandika okubudabuda bosanze mumaka, omu kwomu, mukyama. Bwewabaawo abavubuka emyaka 15 okutuuka ku 17, sooka n’abantu abakulu. | [Checkbox] | Teeka akayini **(√)** mu ka box | | 🡪🡪🡪🡪🡪🡪🡪🡪🡪🡪 | |  | BEGIN | |
| 1.9 | Is this person age 15 to 17? | | Omuntu ono ali wakati w’emyaka 15 ne 17? | Yes…………………. 1  No………….……….. 0 | Yee……………….1  Nedda……………0 | If 1, proceed to 1.10  If 0, proceed to 1.14 | | Bwekiba 1, genda ku namba 1.10  Bweba 0, genda ku 1.14 | | | FIFTEEN |
| 1.10 | | Please obtain oral consent from both the adolescent and the guardian/parent. Start with the adolescents:  Hello, my name is __________. I am a health worker with the Rakai Health Sciences Program helping to support people living in Kasensero.  I would like to provide you with free health counseling and support on preventing and treating HIV. If you agree to participate, I will ask you questions about your health and then counsel you. I will use a special mobile phone tool to help guide the counseling.  We are evaluating this counseling service in a study. You do not have to agree to receive this service and be in the study and can stop participating at any time without any loss of your access to other services provided by the Rakai Health Sciences Program.  We will collect information about you in this study. People at Rakai Health Sciences Program and Johns Hopkins who are involved in the study or who need to make sure the study is being done correctly will see the information.  We will do everything we can to make sure your information is kept private, but there is always a risk of a loss of privacy. This service will also take some of your time and you may be uncomfortable with some of the sensitive questions which I need to ask.  After today’s counseling session, I will try to return to meet with you about every three months for the next several years to continue supporting you. If you are not present when I return and you have a mobile phone contact number, I will try to call or text to see how you are doing.  This service may improve your health and teach you new ways to prevent and treat HIV. This service may also improve the health of the entire Kasensero community.  If you have any questions or concerns about this service and study, call Gertrude Nakigozi (tel 0701444074)  Do you agree to receive this service and participate in the study? | Saba olukusa mubigambo, okuva ew’omuvubuka n’omuzadde oba omuntu amulabirira. Sooka na muvubuka.  Nkulamusizza nyabo/ssebo. Amannya gange nze_______. Nkola ku bya bulamu, wamu n’ekitongole kya Rakai Health Sciences Program, mukuyamba kubyobulamu abantu ababeera mu Kasensero.  Njagala okukuwa okubudabudibwa ku by’obulamu okwobwerere, era okukukwatirako kukuziyiza n’okujjanjaba akawuka ka siriimu. Bwokkiriza okwetaba mu, njakukubuuzaayo ebibuuzo ebikwaata kubulamu bwo,ndyooke nkubudebude. Nja kukozesa essimu eyenjawulo okuyambako mukubudabuda  Twekeneenya okubudabuda kuno, mukunoonyereza kuno. Tolina kukkiriza kwetaba mukufuna kubudabuda oba kwetaba mukunoonyereza, era osobola okubivaamu akaseera konna, awatali kufiirwa bujjanjabi bwofuna okuva mu kitongole kya Rakai Health Sciences Program  Mukunoonyereza kuno, tujja kukubuuza ebikukwatako. Abantu aba Rakai Health Sciences Program ne Johns Hopkins University abenyigidde mukunoonyereza kuno oba abetaaga okulaba nti okunoonyereza kuno kukolebwa bulungi, bajja kulaba ebikukwatako byonompa  Tujja kukola buli ekisoboka okulaba nti byonna ekibukwatako byonotuwa bikuumibwa nga byakyama. Okunoonyereza kuno kujja kutwala kubudde bwo era oyinza obutayisibwa bulungi, ebibuuzo ebimu ebinakubuuzibwa.  Okubudabuda kwaleero nga kuwedde, nja kugezaako okukusisinkana buli luvannyuma lwa myezi esatu okumala emyaka egiwerako mumaaso, okwongera okukuyamba. Bwonoba toliiwo lwenakomawo,singa oba olina akasimu akomungalo, nja kugezaako okukubira ssimu oba okuweereza obubaka ku siimu, okumanya embeera gy’olimu.  Obuweereza buno buyinza okulongoosa embeera y’obulamu bwo n’okukusomesa engeri empya ezokwewala akawuka ka siriimu. Enkola eno eyinza n’okulongoosa embeera y’obulamu bwabantu bomu Kasensero yonna.  Bwoba olina ebibuuzo ebikwata ku kunoonyereza kuno, kuba simu eri Gertrude Nakigozi, ku ssimu 0701444 074  Okkiriza okufuna obuweereza buno obwokubudabuda, n’okwetaba mukunoonyereza kuno? | Yes…………………. 1  No………….……….. 0 | Yee……………….1  Nedda……………0 | | If 1, proceed to 1.11  If 0, proceed to 1.12 | | Bwekiba 1, genda ku namba 1.11  Bweba 0, genda ku 1.12 | CONSENT1 | |
| 1.11 | | Then the parent(s)/guardian:  Hello, my name is __________. I am a health worker with the Rakai Health Sciences Program helping to support people living in Kasensero.  I would like to provide this individual for whom you are the parent or guardian with free health counseling and support on preventing and treating HIV. If you agree to let him or her participate, I will ask him or her some questions, in private, about their health and then counsel them. I will use a special mobile phone tool to help guide the counseling.  We are evaluating this counseling service in a study. You do not have to agree to have this person receive this service and be in the study and they can stop participating at any time without any loss of their access to other services provided by the Rakai Health Sciences Program.  We will collect information about this person in this study. People at Rakai Health Sciences Program and Johns Hopkins who are involved in the study or who need to make sure the study is being done correctly will see the information.  We will do everything we can to make sure their information is kept private, but there is always a risk of a loss of privacy. This service will also take some of their time and they may be uncomfortable with some of the sensitive questions which I need to ask.  This service may improve their health and teach them new ways to prevent and treat HIV. This service may also improve the health of the entire Kasensero community.  After today’s counseling session, I will try to return to meet with them about every three months for the next several years to continue supporting them. If they are not present when I return and they have a mobile phone contact number, I will try to call or text to see how they are doing.  If you have any questions or concerns about this service and study, call Gertrude Nakigozi (tel 0701444074)  Do you agree to allow for this individual for whom you are the parent or guardian to receive this service and participate in this study? | Dddako omuzadde/omulabirizi  Nkulamusizza nyabo/ssebo. Amannya gange nze_______. Nkola ku bya bulamu, wamu n’ekitongole kya Rakai Health Sciences Program, mukuyamba kubyobulamu abantu ababeera mu Kasensero.  Njagala okuwa omwana gw’ozaala oba gw’ovunanyizibwaako, okubudabudibwa ku by’obulamu okwobwerere, era okukukwatirako kukuziyiza n’okujjanjaba akawuka ka siriimu. Bwokkiriza okwetaba mu, njakukubuuzaayo ebibuuzo ebikwaata kubulamu bwo,ndyooke nkubudebude. Nja kukozesa essimu eyenjawulo okuyambako mukubudabuda  Twekeneenya okubudabuda kuno, mukunoonyereza kuno. Tolina kukkiriza muwana gwovunanyizibwaako kufuna kubudabuda oba kwetaba mukunoonyereza, era asobola okubivaamu akaseera konna, awatali kufiirwa bujjanjabi bwafuna okuva mu kitongole kya Rakai Health Sciences Program  Mukunoonyereza kuno, tujja kukubuuza ebikwata kumuntu ono. Abantu aba Rakai Health Sciences Program ne Johns Hopkins University abenyigidde mukunoonyereza kuno oba abetaaga okulaba nti okunoonyereza kuno kukolebwa bulungi, bajja kulaba ebikwata kumuntu oyo, byanatuwa  Tujja kukola buli ekisoboka okulaba nti byonna ekimukwatako byanatuwa bikuumibwa nga byakyama. Okunoonyereza kuno kujja kutwala kubudde bwe era ayinza obutayisibwa bulungi, ebibuuzo ebimu ebinamubuuzibwa.  Okubudabuda kwaleero nga kuwedde, nja kugezaako okumusisinkana buli luvannyuma lwa myezi esatu okumala emyaka egiwerako mumaaso, okwongera okumuyamba. Bwanaba taliiwo lwenakomawo,singa aba alina akasimu akomungalo, nja kugezaako okumubira ssimu oba okuweereza obubaka ku siimu, okumanya embeera gy’alimu.  Obuweereza buno buyinza okulongoosa embeera y’obulamu bwe n’okumusomesa engeri empya ezokwewala akawuka ka siriimu. Enkola eno eyinza n’okulongoosa embeera y’obulamu bwabantu bomu Kasensero yonna.  Bwoba olina ebibuuzo ebikwata ku kunoonyereza kuno, kuba simu eri Gertrude Nakigozi, ku ssimu 0701444 074  Okkiriza omuntu ono okufuna obuweereza buno obwokubudabuda, n’okwetaba mukunoonyereza kuno? | Yes…………………. 1  No………….……….. 0 | Yee……………….1  Nedda……………0 | | If 1, proceed to 2.0  If 0, proceed to 1.12 | | Bwekiba 1, genda ku namba 2.0  Bweba 0, genda ku 1.12 | CONSENT2 | |
| 1.12 | | Thank the person(s) for their time. Please let them know that they can change their mind later and still receive the service. | Webaze omuntu ono olwokukuwa obudde bwe. Mubuulire nti asobola okukyusa endowooza ye oluvannyuma, naye nasigala ng’afuna obujjanjabi | [Checkbox] | Teeka akayini **(√)** mu ka box | | 🡪🡪🡪🡪🡪🡪🡪🡪🡪🡪 | |  | NOCONSEN | |
| 1.13 | | This session is complete. |  | [Button (Save form and close application.)] | Ekitundu kino kiwedde.Tereka byowandiise | | If button pressed, save form and close application. | | Nyiga eppeesa, tereka byowandiise, oggalewo | END4 | |
| 1.14 | | Please obtain oral consent:  Hello, my name is __________. I am a health worker with the Rakai Health Sciences Program helping to support people living in Kasensero.  I would like to provide you with free health counseling and support on preventing and treating HIV. If you agree to participate, I will ask you questions about your health and then counsel you. I will use a special mobile phone tool to help guide the counseling.  We are evaluating this counseling service in a study. You do not have to agree to receive this service and be in the study and can stop participating at any time without any loss of your access to other services provided by the Rakai Health Sciences Program.  We will collect information about you in this study. People at Rakai Health Sciences Program and Johns Hopkins who are involved in the study or who need to make sure the study is being done correctly will see the information.  We will do everything we can to make sure your information is kept private, but there is always a risk of a loss of privacy. This service will also take some of your time and you may be uncomfortable with some of the sensitive questions which I need to ask.  This service may improve your health and teach you new ways to prevent and treat HIV. This service may also improve the health of the entire Kasensero community.  After today’s counseling session, I will try to return to meet with you about every three months for the next several years to continue supporting you. If you are not present when I return and you have a mobile phone contact number, I will try to call or text to see how you are doing.  If you have any questions or concerns about this service and study, call Gertrude Nakigozi (tel 0701444074)  Do you agree to receive this service and participate in the study? Yes ___ No ____ | Saba olukusa mubigambo, okuva ew’omuvubuka n’omuzadde oba omuntu amulabirira. Sooka na muvubuka.  Nkulamusizza nyabo/ssebo. Amannya gange nze_______. Nkola ku bya bulamu, wamu n’ekitongole kya Rakai Health Sciences Program, mukuyamba kubyobulamu abantu ababeera mu Kasensero.  Njagala okukuwa okubudabudibwa ku by’obulamu okwobwerere, era okukukwatirako kukuziyiza n’okujjanjaba akawuka ka siriimu. Bwokkiriza okwetaba mu, njakukubuuzaayo ebibuuzo ebikwaata kubulamu bwo,ndyooke nkubudebude. Nja kukozesa essimu eyenjawulo okuyambako mukubudabuda  Twekeneenya okubudabuda kuno, mukunoonyereza kuno. Tolina kukkiriza kwetaba mukufuna kubudabuda oba kwetaba mukunoonyereza, era osobola okubivaamu akaseera konna, awatali kufiirwa bujjanjabi bwofuna okuva mu kitongole kya Rakai Health Sciences Program  Mukunoonyereza kuno, tujja kukubuuza ebikukwatako. Abantu aba Rakai Health Sciences Program ne Johns Hopkins University abenyigidde mukunoonyereza kuno oba abetaaga okulaba nti okunoonyereza kuno kukolebwa bulungi, bajja kulaba ebikukwatako byonompa  Tujja kukola buli ekisoboka okulaba nti byonna ekibukwatako byonotuwa bikuumibwa nga byakyama. Okunoonyereza kuno kujja kutwala kubudde bwo era oyinza obutayisibwa bulungi, ebibuuzo ebimu ebinakubuuzibwa.  Okubudabuda kwaleero nga kuwedde, nja kugezaako okukusisinkana buli luvannyuma lwa myezi esatu okumala emyaka egiwerako mumaaso, okwongera okukuyamba. Bwonoba toliiwo lwenakomawo,singa oba olina akasimu akomungalo, nja kugezaako okukubira ssimu oba okuweereza obubaka ku siimu, okumanya embeera gy’olimu.  Obuweereza buno buyinza okulongoosa embeera y’obulamu bwo n’okukusomesa engeri empya ezokwewala akawuka ka siriimu. Enkola eno eyinza n’okulongoosa embeera y’obulamu bwabantu bomu Kasensero yonna.  Bwoba olina ebibuuzo ebikwata ku kunoonyereza kuno, kuba simu eri Gertrude Nakigozi, ku ssimu 0701444 074  Okkiriza okufuna obuweereza buno obwokubudabuda, n’okwetaba mukunoonyereza kuno?  Yee_________Nedda_________ | Yes…………………. 1  No………….……….. 0 | Yee……………….1  Nedda……………0 | | If 1, proceed to 2.0  If 0, back to 1.12 | | Bwekiba 1, genda ku namba 2.0  Bweba 0, ddayo ku namba 1.12 | CONSENT4 | |

| **Individual Screening-**performed client by client, in private | | | | | | | | | | | |
| --- | --- | --- | --- | --- | --- | --- | --- | --- | --- | --- | --- |
| 2.0 | | O Let the client know that you need to start by asking some important questions.  O All of the information they share will be kept private.  O There are no wrong or right answers. You are just trying to find out how best to help them. | O Omuntu gwokolako mutegeeze nti ogenda kusooka kumubuuzayo ebibuuzo.  O Byonna by’anakubuulira bijja kukuumibwa nga byakyaama  .  O Tewali kiddibwamu kikyamu oba kituufu. Ogezaako kunoonya ngeri esinga eyokumuyambamu. | [Checkbox] | Teeka akayini **(√)** mu ka box | | 🡪🡪🡪🡪🡪🡪🡪🡪🡪🡪 | |  | | IS1 |
| 2.1 | | Have you ever participated in this counseling service before? | Wali wetabye mukubudabudibwa kuno? | Yes…………………. 1  No………………….. 0  Not sure……………. 77 | Yee…………………. 1  Nedda………………….. 0  Sekakasa………………77 | | If 1, proceed to 2.3  If 0 or 77, 🡪🡪🡪🡪🡪🡪🡪 | |  | | NEW2 |
| 2.2 | | Begin a new Log Book sheet for this participant. The Participant ID is [Display Participant ID] |  | [Checkbox] | Teeka akayini **(√)** mu ka box | | Proceed to 2.4 | |  | | NEWLOG |
| 2.3 | | Locate the Log Book sheet for this participant. The Participant ID is [Display Participant ID] |  | [Checkbox] | Teeka akayini **(√)** mu ka box | | 🡪🡪🡪🡪🡪🡪🡪🡪🡪🡪 | |  | | OLDLOG |
| 2.4 | | Age | Olina emyaka emeka? | [Number] | [Ennamba]_________ | | 🡪🡪🡪🡪🡪🡪🡪🡪🡪🡪 | |  | | AGE |
| 2.5 | | Gender | Musajja oba mukazi? | Male…………………. 1  Female………………… 2 | Musajja………………. 1  Mukazi…………………..2 | | If 1, suppress question 2.15  If 2, suppress question 2.14 | |  | | SEX |
| 2.6 | | Marital Status | Embeera y’obufumbo bwe | Single………………. 1  Divorced…………… 2  Widowed…………... 3  Married (Non-polygamous)….…… 4  Married (Polygamous).. 5  Other……………….. 6 | Simufumbo……………1  Yayawukana n’omwagalwa we…………………………2  Yafiirwa………….............3  Mufumbo (alina omwagalwa omu?……….4  Mufumbo (alina abagalwa abasukka mw’omu)….. 5  Ekirala……………………6 | | 🡪🡪🡪🡪🡪🡪🡪🡪🡪🡪 | |  | | MARITAL |
| 2.7 | | Occupations (may select more than one). | Okola mulimu/mirimu ki? (osobola okulondako ogusukka mugumu). | Fishing on the Lake…. 1  Fish factory worker….. 2  Agriculture………. 3 Housework in your  own home………….. 4  Housekeeper (for  relative or employer) … 5 Home brewing……… 6  Government/clerical.. 7  Teacher……. 8  Student….. 9 Military/police…… 10 Shopkeeper….. 11 Trading/vending….. 12 Bar worker or owner…. 13 Trucker/Transport…. 14  Other……. 15 | Avuba kunnyanja………1  Akola mu fakitole yebyennyanja………….2  Mulimi…………………...3 Akola mirimu gyamumaka ge………………………4  Akola mirimu gyamumaka gamuntu mulala (owoluganda oba omulala)………………..5 Afumba mwenge awaka ..6  Akola mirimu gya gavumenti……………….7  Musomesa……………….8  Muyizi…………………….9 Musirikale/mupolisi…… 10 Atunda duuka………….11 Atunda byamaguzi/ musuubuzi……………..12 Alina oba akola mu bbaala………………….. 13 Dereva oba avuga bimotoka ebinene…. 14  Ekirala………………….15 | | 🡪🡪🡪🡪🡪🡪🡪🡪🡪🡪 | |  | | OCCUP |
| 2.8 | | Have you ever been tested for HIV? | Wali wekebezza akawuka ka siriimu? | Yes…………………. 1  No………………….. 0  Not sure……………. 77  Declined to answer.. 88 | Yee…………………. 1  Nedda………………….. 0  Sikakasa……………. 77  Agaanye okuddamu…..88 | | If 0, then skip to 2.14 OR 2.15  Else, 🡪🡪🡪🡪🡪🡪 | | Bwekiba “***Nedda’,*** buuka odde ku nnamba 2.12 | | TESTED |
| 2.9 | | Have you been HIV tested in the past 12 months? | Wekebezzaako akawuka ka siriimu mu myezi ekkumi n’ebiri egiyise? | Yes…………………. 1  No………………….. 0  Not sure……………. 77  Declined to answer.. 88 | Yee…………………. 1  Nedda………………….. 0  Sikakasa……………. 77  Agaanye okuddamu…...88 | | 🡪🡪🡪🡪🡪🡪🡪🡪🡪🡪 | |  | | TESTED12 |
| 2.10 | | Have you ever tested positive for HIV? | Ebiva mukukebera kw’omusaayi gwo byali biraze nti olina akawuka ka siriimu? | Yes…………………. 1  No………………….. 0  Not sure……………. 77  Declined to answer.. 88 | Yee…………………. 1  Nedda………………….. 0  Sikakasa……………….. 77  Agaanye okuddamu…..88 | | If 0, then skip to 2.14 OR 2.15  Else, 🡪🡪🡪🡪🡪🡪 | | Bwekiba “***Nedda’,*** buuka odde ku nnamba 2.12 | | HIVPOS |
| 2.11 | | Are you taking Septrin? | Omira septrin? | Yes…………………. 1  No………………….. 0  Not sure……………. 77  Declined to answer.. 88 | Yee…………………. 1  Nedda………………….. 0  Sikakasa……………. 77  Agaanye okuddamu…...88 | | 🡪🡪🡪🡪🡪🡪🡪🡪🡪🡪 | |  | | TAKESEP |
| 2.12 | | Are you taking antiretrovirals for HIV? | Omira eddagala erikendeeza obungi bwakawuka ka siriimu? (ARVs) okujjanjaba akawuka ka siriimu? | Yes…………………. 1  No………………….. 0  Not sure……………. 77  Declined to answer.. 88 | Yee…………………. 1  Nedda………………….. 0  Sikakasa……………. 77  Agaanye okuddamu…...88 | | 🡪🡪🡪🡪🡪🡪🡪🡪🡪🡪 | |  | | TAKEART |
| 2.13 | | When did you last go to a clinic or hospital for HIV care? | Wasemba ddi okugenda ku ddwaliro/akalwaliro okufuna obujjanjabi bwakawuka ka siriimu? | Within the past 6  months…………. 1  Between 6-12 months.. 2  Over 12 months ago.. 3  Never gone…... 4  Not sure….. 77  Declined to answer.. 88 | Mu myezi omukaaga egiyise…………………..1  Wakati w’emyezi mukaaga nekkumi n’ebiri…………2  Omwaka (emyezi kkumi n’ebiri) n’okuingawo……3  Sigendanga…...............4  Sekakasa………………77  Agaanye okuddamu…..88 | | 🡪🡪🡪🡪🡪🡪🡪🡪🡪🡪 | |  | | CARE |
| 2.14 | | Are you circumcised? | Wakomolebwa? | Yes…………………. 1  No………………….. 0  Not sure……………. 77  Declined to answer.. 88 | Yee…………………. 1  Nedda………………….. 0  Sikakasa……………. 77  Agaanye okuddamu…...88 | | Skip to 2.16 | |  | | CIRC |
| 2.15 | | Are you pregnant? | Olina olubuto? | Yes…………………. 1  No………………….. 0  Not sure……………. 77 Declined to answer.. 88 | Yee…………………. 1  Nedda………………….. 0  Sikakasa……………. 77  Agaanye okuddamu…...88 | | 🡪🡪🡪🡪🡪🡪🡪🡪🡪🡪 | |  | | PREG |
| 2.16 | | Have you had sex without a condom in the past 12 months? | Mu myezi ekkumi n’ebiri egiyise, wegasseko n’omuntu yenna nga tokozesezza kapiira? | Yes…………………. 1  No………………….. 0  Not sure……………. 77  Declined to answer.. 88 | Yee…………………. 1  Nedda………………….. 0  Sikakasa……………. 77  Agaanye okuddamu…...88 | | 🡪🡪🡪🡪🡪🡪🡪🡪🡪🡪 | |  | | CONDOM |
| 2.17 | | Over the last year (12 months), how much time do you spend staying outside of Kansensero? | Mu myezi ekkumi n’ebiri egiyise, omaze ekiseera kyenkanawa ng’obeera bweru wa Kasensero? | Most of the year….. 1  About half of the year.. 2  A few months……. 3  A few weeks……… 4  A few days…….. 5  Never gone……… 6  Declined to answer.. 88 | Ekiseera ekisinga….. 1  Nga kitundu kyamwaka.. 2  Emyezi mitono……. 3  Wiki ntono……… 4  Ennaku ntono…….. 5  Sigendanga….............. 6  Agaanye okuddamu…..88 | | 🡪🡪🡪🡪🡪🡪🡪🡪🡪🡪 | |  | | MOBILITY |
| 2.18 | | Do you have any children? | Olina omwana/abaana? | Yes…………………. 1  No………………….. 0  Declined to answer.. 88 | Yee…………………. 1  Nedda………………….. 0  Agaanye okuddamu…..88 | | 🡪🡪🡪🡪🡪🡪🡪🡪🡪🡪 | |  | | CHILD |
|  | **Module Activation Algorithms**-based on responses above, the relevant HIV risk modules are activated below. | | | | | | | | | | |
| A | IF [[2.8=(0 OR 77 OR 88) OR 2.9=(0 OR 77 OR 88)] AND [2.5=1 OR 2.15=(0)]] THEN activate Module A | | | | | | | | | | |
|  | IF [[“Have you ever been tested for HIV” is No (0) or Not Sure (77) or Declined to Answer (88) OR “Have you been HIV tested in the last 12 months” is No (0) or Not Sure (77) or Declined to Answer (88)] AND “Have you ever tested positive for HIV” is No (0) or Not Sure (77) or Declined to Answer (88) AND “Are you pregnant” is No (0)] THEN activate Module A (HIV serostatus unknown or no recent HIV test, Male or Female not pregnant) | | | | | | | | | | |
| B | IF [2.8=[(0 OR 77 OR 88) OR 2.9=(0 OR 77 OR 88)] AND 2.15=(1 OR 77 OR 88)] THEN activate Module B | | | | | | | | | | |
|  | IF [“Have you ever been tested for HIV” is No (0) or Not Sure (77) or Declined to Answer (88) OR “Have you been HIV tested in the last 12 months” is No (0) or Not Sure (77) or Declined to Answer (88) AND “Have you ever tested positive for HIV” is No (0) or Not Sure (77) or Declined to Answer (88) AND “Are you pregnant” is Yes (1) or Not Sure (77) or Declined to Answer (88)] THEN activated Module B (HIV serostatus unknown or no recent HIV test, Female pregnant) | | | | | | | | | | |
| C | IF [2.5=1 AND 2.14=(0 OR 77 OR 88)] THEN activate Module C | | | | | | | | | | |
|  | IF [Gender is Male (1) AND “Are you circumcised” is No (0) or Not Sure (77) or Declined to Answer (88)] THEN activate Module C (Male, MMC-) | | | | | | | | | | |
| D | IF [[2.10=1 AND [2.11=(0 OR 77 OR 88) OR 2.12=(0 OR 77 OR 88)] AND 2.13=(2 OR 3 OR 4 OR 77 OR 88)]] THEN activate Module D | | | | | | | | | | |
|  | IF [“Have you ever tested positive for HIV” is Yes (1) AND “Are you taking Septrin” is No (0) or Not Sure (77) or Declined to Answer (88) OR “Are you taking antiretrovirals for HIV” is No (0) or Not Sure (77) or Declined to Answer (88) OR “When did you last go to a clinic or hospital for HIV care” is Between 6 and 12 Months Ago (2) or Over 12 Months Ago (3) or Never Gone (4) or Not Sure (77) or Declined to Answer (880] THEN activate Module D (HIV-positive, Not in care) | | | | | | | | | | |
| E | IF [2.10=1 AND 2.12=(0 OR 77 OR 88) AND 2.13=1] THEN activate Module E | | | | | | | | | | |
|  | IF [“Have you ever tested positive for HIV” is Yes (1) AND “Are you taking antiretrovirals for HIV” is No (0) or Not Sure (77) or Declined to Answer (88) AND “When did you last go to a clinic or hospital for HIV care” is Within the past 6 months (1)] THEN activate Module E (HIV-positive, In care, Not on ART) | | | | | | | | | | |
| F | IF [2.10=1 AND 2.12=1] THEN activate Module F | | | | | | | | | | |
|  | IF [“Have you ever tested positive for HIV” is Yes (1) AND “Are you taking antiretrovirals for HIV” is Yes (1)] THEN activate Module F (HIV-positive, On ART) | | | | | | | | | | |
| G | IF [2.15=(1 OR 77 OR 88) AND 2.10=1] THEN activate Module G | | | | | | | | | | |
|  | IF [“Are You Pregnant” is Yes (1) Not Sure (77) or Declined to Answer (88) AND “Have you ever tested positive for HIV” is Yes (1)] THEN Activate Module G (Pregnant +, HIV +) | | | | | | | | | | |
| H | IF [2.5=1 AND 2.16=(1 OR 77 OR 88)] THEN activate Module H | | | | | | | | | | |
|  | IF [Gender is Male (1) AND “Have you had sex without a condom in the last 12 months” is Yes (1) or Not Sure (77) or Declined to Answer (88)] THEN activate module H(Risky Sex+, Male+) | | | | | | | | | | |
| I | IF [2.5=2 AND 2.16=(1 OR 77 OR 88)] THEN activate Module I | | | | | | | | | | |
|  | IF {Gender is Female (2) AND “Have you had sex without a condom in the last 12 months” is Yes (1) or Not Sure (77) or Declined to Answer (88)] THEN activate Module I (Risky Sex+, Female+) | | | | | | | | | | |
| JK | ALWAYS activate Modules J and K | | | | | | | | | | |
|  | **MODULES**-modules are activated based on responses to individual screening questions above. | | | | | | | | | | |
| **A** | | **HIV serostatus unknown or no recent HIV test, Male or Female not pregnant** | Embeera y’akawuka ka siriimu temanyiddwa, oba teyekebeza kawuka ka siriimu mubiseera ebiyise; omusajja oba omukazi atalina lubuto |  | |  | |  | |  |  |
| 3.1 | | Please ask the client the following questions using the motivational interviewing skills you have learned. | Buuza ebibuuzo bino wammanga ng’okozesa obukugu obuzzaamu amaanyi mukubuuza ebibuuzo bw’oyize |  | |  | | 🡪🡪🡪🡪🡪🡪🡪🡪🡪🡪 | |  |  |
| 3.2 | | **Information**  O *Can you tell me what you know about HIV testing?* | O *Osobola okumbuulira by’omanyi ku kwekebeza akawuka ka siriimu?* | [Checkbox] | | Teeka akayini **(√)** mu ka box | | 🡪🡪🡪🡪🡪🡪🡪🡪🡪🡪 | |  | **AINFO1** |
| 3.3 | | **Motivation**  *O What would be some benefits to getting an HIV test?*  *O What are your concerns about getting an HIV test?*  *O What have been the not so good things that have happened or may happen by not getting tested?* | *O Birungi ki ebiva mukwekebeza akawuka ka siriimu?*  *O Biki byeweralikirira ku kwekebeza akawuka ka siriimu?*  *O Bintu ki ebitali birungi ebibaddewo oba ebiyinza okubaawo singa omuntu teyekebeza?* | [Checkbox] | | Teeka akayini **(√)** mu ka box | | 🡪🡪🡪🡪🡪🡪🡪🡪🡪🡪 | |  | **AMOTI1** |
| 3.4 | | **Behavioral Skills**  O *How confident are you that you could get an HIV test if you wanted to?*    *O What would make getting the test easy for you to do if you wanted to?*  *O What would make getting the test difficult for you to do if you wanted to?* | *O Oli mukakafu kwenkanawa nti osobola okwekebeza akawuka ka siriimu singa oba oyagadde?*  *O Kiki ekiyinza okukwanguyiza okwekebeza akawuka ka siriimu singa oba oyagadde?*  *O Kiki ekiyinza okukuzibuwaliza okwekebeza akawuka ka siriimu singa oba oyagadde?* | [Checkbox] | | Teeka akayini **(√)** mu ka box | | 🡪🡪🡪🡪🡪🡪🡪🡪🡪🡪 | |  | **ABEHA1** |
| 3.5 | | **Intentions**  *O Given how you feel right now, would you want to get HIV tested within the next month?* | *O Okusinziira bwewewulira kati, wandiyagadde okwekebeza akawuka ka siriimu mu bbanga eritasussa mwezi gumu mu maaso?* | Yes…………………. 1  No………………….. 0  Not sure……………. 77 | | Yee…………………. 1  Nedda………………….. 0  Sikakasa……………….77 | | If 1, then skip to 3.7  Else, 🡪🡪🡪🡪🡪🡪 | | Bwekiba Yee, buuka ogende ku namba 3.7 | **AMOTI2** |
| 3.6 | | **Targets**  *O What would need to change for you to want to get tested?* | *O Kiki kyewandyetaaze okukyusa okukusobozesa okwagala okwekebeza akawuka ka siriimu?* | [Checkbox] | | Teeka akayini **(√)** mu ka box | | 🡪🡪🡪🡪🡪🡪🡪🡪🡪🡪 | |  | **AMOTI3** |
| 3.7 | | *Some people are ready to get tested some are not ready right now. Most people think that it is helpful to have information, no matter how ready they are.*  *So if it is okay with you, I will share some additional information with you:*    O HIV Testing is free. **[I]** O The RHSP Kasensero clinic is the nearest HIV testing location.**[I]** O Support is available, myself and others can support and help you. **[M]** O Facilitated disclosure is available if needed. **[B]** | *Abantu abamu betegefu okwekebeza, abamu sibetegefu kati.Abantu abasinga balowooza nti kyamugaso okufuna okumanyisibwa, nebwebaba betegefu oba nedda. N’olwekyo, bwekiba tekirina buzibu, nja kugabana naawe ebintu bino wammanga*  O Okukebera akawuka ka siriimu kwabwereere. **[I]** O Akalwaliro ka RHSP e Kasensero wewakebererwa akawuka ka siriimu awasinga okuba okumpi..**[I]** O Obuyambi webuli. Nze n’abantu abalala tusobola okukuyamba. **[M]** O Obuyambi bwokubuulira omuntu oba abantu bo nti olina akawuka ka siriimu webuli singa oba obwetaaze.d. **[B]** | [Checkbox] | | Teeka akayini **(√)** mu ka box | | 🡪🡪🡪🡪🡪🡪🡪🡪🡪🡪 | |  | **A1** |
| **B** | | **HIV serostatus unknown or no recent HIV test, Female pregnant** | **Embeera y’akawuka ka siriimu temanyiddwa, oba teyekebeza kawuka ka siriimu mubiseera ebiyise; omukazi alina lubuto** |  | |  | |  | |  |  |
| 4.1 | | Please ask the client the following questions using the motivational interviewing skills you have learned. | Buuza ebibuuzo bino wammanga ng’okozesa obukugu obuzzaamu amaanyi mukubuuza ebibuuzo bw’oyize |  | |  | | 🡪🡪🡪🡪🡪🡪🡪🡪🡪🡪 | |  |  |
| 4.2 | | **Information**  O *Can you tell me what you know about HIV testing?*  O *Can you tell me what you know about HIV testing when a woman is pregnant?* | O *Osobola okumbuulira by’omanyi ku kwekebeza akawuka ka siriimu?*  O *Osobola okumbuulira by’omanyi ku kwekebeza akawuka ka siriimu omukyala ng’ali lubuto?* | [Checkbox] | | Teeka akayini **(√)** mu ka box | | 🡪🡪🡪🡪🡪🡪🡪🡪🡪🡪 | |  | **BINFO1** |
| 4.3 | | **Motivation**  *O What would be some benefits to getting an HIV test?*  *O What would be some benefits to getting an HIV test when pregnant?*  *O What are your concerns about getting an HIV test?*  *O What have been the not so good things that have happened or may happen by not getting tested?* | *O Birungi ki ebiva mukwekebeza akawuka ka siriimu?*  O *Birungi ki ebiva mukwekebeza akawuka ka siriimu ng’omukyala alina olubuto?*  *O Biki byeweralikirira ku kwekebeza akawuka ka siriimu?*  *O Bintu ki ebitali birungi ebibaddewo oba ebiyinza okubaawo singa omuntu teyekebeza?* | [Checkbox] | | Teeka akayini **(√)** mu ka box | | 🡪🡪🡪🡪🡪🡪🡪🡪🡪🡪 | |  | **BMOTI1** |
| 4.4 | | **Behavioral Skills**  O *How confident are you that you could get an HIV test if you wanted to?*    *O What would make getting the test easy for you to do if you wanted to?*  *O What would make getting the test difficult for you to do if you wanted to?* | *O Oli mukakafu kwenkanawa nti osobola okwekebeza akawuka ka siriimu singa oba oyagadde?*  *O Kiki ekiyinza okukwanguyiza okwekebeza akawuka ka siriimu singa oba oyagadde?*  *O Kiki ekiyinza okukuzibuwaliza okwekebeza akawuka ka siriimu singa oba oyagadde?* | [Checkbox] | | Teeka akayini **(√)** mu ka box | | 🡪🡪🡪🡪🡪🡪🡪🡪🡪🡪 | |  | **BBEHA1** |
| 4.5 | | **Intentions**  *O Given how you feel right now, would you want to get HIV tested within the next month?* | *O Okusinziira bwewewulira kati, wandiyagadde okwekebeza akawuka ka siriimu mu bbanga eritasussa mwezi gumu mu maaso?* | Yes…………………. 1  No………………….. 0  Not sure……………. 77 | | Yee…………………. 1  Nedda………………….. 0  Sikakasa…………… …77 | | If 1, then skip to 4.7  Else, 🡪🡪🡪🡪🡪🡪 | | Bwekiba Yee, buuka ogende ku namba 4.7 | **BMOTI2** |
| 4.6 | | **Targets**  *O What would need to change for you to want to get tested?* | *O Kiki kyewandyetaaze okukyusa okukusobozesa okwagala okwekebeza?* | [Checkbox] | |  | | 🡪🡪🡪🡪🡪🡪🡪🡪🡪🡪 | |  | **BMOTI3** |
| 4.7 | | *Some people are ready to get tested some are not ready right now. Most people think that it is helpful to have information, no matter how ready they are.*  *So if it is okay with you, I will share some additional information with you:*    O HIV Testing is free. **[I]** O The RHSP Kasensero clinic is the nearest HIV testing location.**[I]** O Support is available, myself and others can support and help you. **[M]** O Facilitated disclosure is available if needed. **[B]**  O For pregnant women, getting tested for HIV is a great step to making sure they have a healthy baby. **[I,M]** | *Abantu abamu beetegefu okwekebeza, abamu sibetegefu kati.Abantu abasinga balowooza nti kyamugaso okufuna okumanyisibwa, nebwebaba betegefu oba nedda. N’olwekyo, bwekiba tekirina buzibu, nja kugabana naawe ebintu bino wammanga*  O Okukebera akawuka ka siriimu kwabwereere. **[I]** O Akalwaliro ka RHSP e Kasensero wewakebererwa akawuka ka siriimu awasinga okuba okumpi**..[I]** O Obuyambi webuli. Nze n’abantu abalala tusobola okukuyamba. **[M]** O Obuyambi bwokubuulira omuntu oba abantu bo nti olina akawuka ka siriimu webuli singa oba obwetaaze.d. **[B]**  O Abakyala abembuto, okwekebeza akawuka ka siriimu kikolwa kikulu nnyo eri okukakasa nti bazaala omwana omulamu **[I,M]** | [Checkbox] | | Teeka akayini **(√)** mu ka box | | 🡪🡪🡪🡪🡪🡪🡪🡪🡪🡪 | |  | **B1** |
| **C** | | **Male, MMC-** | **Okukomola kw’abaami** |  | |  | |  | |  |  |
| 5.1 | | Please ask the client the following questions using the motivational interviewing skills you have learned. | Buuza ebibuuzo bino wammanga ng’okozesa obukugu obuzzaamu amaanyi mukubuuza ebibuuzo bw’oyize |  | |  | | 🡪🡪🡪🡪🡪🡪🡪🡪🡪🡪 | |  |  |
| 5.2 | | **Information**  O *Can you tell me what you know about MMC?* | O *Osobola okumbuulira by’omanyi kukukomolebwa kwabasajja?* | [Checkbox] | | Teeka akayini **(√)** mu ka box | | 🡪🡪🡪🡪🡪🡪🡪🡪🡪🡪 | |  | **CINFO1** |
| 5.3 | | **Motivation**  *O What would be some benefits of MMC?*  *O What are your concerns about circumcision?*  *O What have been the not so good things that have happened or may happen by not being circumcised?* | *O Birungi ki ebiri mukukomola kw’abasajja?*  *O Biki byeweralikirira ku kukomola kwabasajja?*  *O Bintu ki ebitabadde birungi ebibaddewo oba ebiyinza okubaawo singa omusajja takomolebwa?* | [Checkbox] | | Teeka akayini **(√)** mu ka box | | 🡪🡪🡪🡪🡪🡪🡪🡪🡪🡪 | |  | **CMOTI1** |
| 5.4 | | **Behavioral Skills**  O *How confident are you that you could get MMC if you wanted to?*  *O What are things that would make it difficult to get MMC even if you wanted to?* | *O Oli mukakafu kwenkanawa nti osobola okufuna okukomolebwa singa oba oyagadde?*  *O Bintu ki ebiyinza okuzibuwaza okufuna okukomolebwa nebwewandibadde okwagadde?* | [Checkbox] | | Teeka akayini **(√)** mu ka box | | 🡪🡪🡪🡪🡪🡪🡪🡪🡪🡪 | |  | **CBEHA1** |
| 5.5 | | **Intentions**  *O Given how you feel right now, would you want MMC within the next month?* | *O Okusinziira bw’owulira kati, wandyagadde okukomolebwa mubbanga eritasussa mwezi gumu?* | Yes…………………. 1  No………………….. 0  Not sure……………. 77 | | Yee…………………. 1  Nedda………………….. 0  Sikakasa…………… …77 | | If 1, then skip to 5.7  Else, 🡪🡪🡪🡪🡪🡪 | | Bwekiba Yee, buuka ogende ku namba 5.7 | **CMOTI2** |
| 5.6 | | **Targets**  *O What would need to change for you to want to get MMC?* | *O Kiki ekyandikyuseemu okusobola okukwagaza okukomolebwa?* | [Checkbox] | | Teeka akayini **(√)** mu ka box | | 🡪🡪🡪🡪🡪🡪🡪🡪🡪🡪 | |  | **CMOTI3** |
| 5.7 | | *Some people are ready to get circumcised and some are not ready right now. Most people think that it is helpful to have information, no matter how ready they are. So if it is okay with you, I will share some additional information with you:*  O If you are concerned about pain, we now have some new ways to minimize pain. **[I]** | Abantu abamu beetegefu kukomolebwa ate abamu sibetegefu. *Abantu abasinga balowooza nti kyamugaso okufuna okumanyisibwa, nebwebaba betegefu oba nedda. N’olwekyo, bwekiba tekirina buzibu, nja kugabana naawe ebintu bino wammanga*  O Bwoba mweralikirivu ku kufuna obulumi, kati tulina engeri empya ekendeeza obulumi. **[I]** | [Checkbox] | | Teeka akayini **(√)** mu ka box | | 🡪🡪🡪🡪🡪🡪🡪🡪🡪🡪 | |  | **C1** |
| **D** | | **HIV-positive, Not in care** | **Abalina akawuka ka siriimu naye nga tebafuna bujjanjabi** |  | |  | |  | |  |  |
| 6.1 | | Please ask the client the following questions using the motivational interviewing skills you have learned. | Buuza ebibuuzo bino wammanga ng’okozesa obukugu obuzzaamu amaanyi mukubuuza ebibuuzo bw’oyize. |  | |  | | 🡪🡪🡪🡪🡪🡪🡪🡪🡪🡪 | |  |  |
| 6.2 | | **Information**  O *Can you tell me what you know about HIV care?*  O *What do you know about the availability of HIV care when you are not staying in Kasensero?* | O Osobola okumbuulira by’omanyi kubujjanjabi bw’akawuka ka siriimu?  O Kiki ky’omanyi kubujjanjabi  bw’akawuka ka siriimu ng’a tobeera Kasensero? | [Checkbox] | | Teeka akayini **(√)** mu ka box | | 🡪🡪🡪🡪🡪🡪🡪🡪🡪🡪 | |  | **DINFO1** |
| 6.3 | | **Motivation**  *O What would be some benefits to getting into HIV care?*  *O What are your concerns about HIV care?*  *O What are your concerns about getting health care when you are not staying in Kasensero?*  *O What have been the not so good things that have happened or may happen by not getting into care?* | *O Birungi ki ebiri mukufuna obujjanjabi bwa kawuka ka siriimu?*  *O Bweralikirivu ki bwolina kubujjanjabi bw’akawuka ka siriimu?*  *O Bweralikirivu ki bwolina kukufuna kubujjanjabi bw’akawuka ka siriimu nga tobeera Kasensero?*  *O Biki ebitabadde birungi ebibaddewo oba ebiyinza okubaawo singa omuntu tafuna bujjanjabi bwa kawuka ka siriimu?* | [Checkbox] | | Teeka akayini **(√)** mu ka box | | 🡪🡪🡪🡪🡪🡪🡪🡪🡪🡪 | |  | **DMOTI1** |
| 6.4 | | **Behavioral Skills**  O *How confident are you that you could get HIV care if you wanted to?*  *O What are things that would make it difficult to get HIV care even if you wanted to?* | *O Oli mukakafu kwenkanawa nti osobola okufuna obujjanjabi bwa kawuka ka siriimu singa oba obwetaaze?*  *O Bintu ki ebiyinza okuzibuwaza okufuna obujjanjabi bwa kawuka ka siriimu, nebwoba oyagadde okubufuna?* | [Checkbox] | | Teeka akayini **(√)** mu ka box | | 🡪🡪🡪🡪🡪🡪🡪🡪🡪🡪 | |  | **DBEHA1** |
| 6.5 | | **Intentions**  *O Given how you feel right now, would you want to get HIV care?*  *O Given how you feel right now, do you plan on getting HIV care when you travel?* | *O Okusinziira bwewewulira kati, wandyagadde okufuna obujjanjabi bwakawuka ka siriimu?*  *O Okusinziira bwewewulira kati, oteekateeka okufuna obujjanjabi bwakawuka ka siriimu ng’otambudde?* | Yes…………………. 1  No………………….. 0  Not sure……………. 77  Yes…………………. 1  No………………….. 0  Not sure……………. 77 | | Yee…………………. 1  Nedda………………….. 0  Sekakasa……………. 77  Yee…………………. 1  Nedda………………….. 0  Sekakasa……………. 77 | | If 1 for both, then skip to 6.7  Else, 🡪🡪🡪🡪🡪🡪 | | Bwekiba Yee, buuka ogende ku namba 6.7 | **DMOTI2**  **DMOTI2B** |
| 6.6 | | **Targets**  *O What would need to change for you to want to get HIV care either in Kasensero or when traveling?* | *O Biki ebyetaaga okukyuuka okukusobozesa okwagala okufuna obujjanjabi bw’akawuka ka siriimu ng’oli e Kasensero oba ng’otambudde?* | [Checkbox] | | Teeka akayini **(√)** mu ka box | | 🡪🡪🡪🡪🡪🡪🡪🡪🡪🡪 | |  | **DMOTI3** |
| 6.7 | | *Some people are ready to get in care and some are not ready right now. Most people think that it is helpful to have information, no matter how ready they are. So if it is okay with you, I will share some additional information with you:*  O HIV Care is free at the RHSP clinic. **[I]**  O If you need a referral to another health clinic while you are traveling, I can help you. **[I]**  O If you have children, having them tested for HIV is a great step to making sure they are healthy. **[I,M]** | Abantu abamu betegefu okufuna obujjanjabi bw’akawuka ka siriimu, ate abalala sibetegefu. Abantu abasinga balowooza nti kyamugaso okufuna okumanyisibwa, nebwebaba betegefu oba nedda. Bwekiba tekirina buzibu, nja kugabana naawe ebintu bino wammanga  O Obujjajnjabi bw’akawuka ka siriimu bwabwereere ku kalwaliro ka RHSP. **[I]**  O Bwoba wetaaga okuweerezebwa ku kilinika endala, ng’obadde otambudde, nsobola okukuyamba **[I]**  O Bwoba olina abaana, kikulu nnyo okubakebeza akawuka ka siriimu, okukakasa nti balamu . **[I,M]** | [Checkbox] | | Teeka akayini **(√)** mu ka box | | 🡪🡪🡪🡪🡪🡪🡪🡪🡪🡪 | |  | **D1** |
| **E** | | **HIV-positive, In care, Not on ART** | **Abalina akawuka ka siriimu, nga bafuna obujjanjabi naye nga tebali ku ddagala lya ARV** |  | |  | |  | |  |  |
| 7.1 | | Please ask the client the following questions using the motivational interviewing skills you have learned. | Buuza ebibuuzo bino wammanga ng’okozesa obukugu obuzzaamu amaanyi mukubuuza ebibuuzo bw’oyize. |  | |  | | 🡪🡪🡪🡪🡪🡪🡪🡪🡪🡪 | |  |  |
| 7.2 | | **Information**  O *Can you tell me what you know about ART?*  O *What do you know about the availability of ART when you are not staying in Kasensero?* | O *Osobola okumbuulira by’omanyi ku ddagala erikendeeza obungi bwakawuka mumubiri (ARV)?*  O Kiki ky’omanyi ku *wasobola okufunibwa eddagala erikendeeza obungi bwakawuka mumubiri (ARVs), nga tobeera mu Kasensero?* | [Checkbox] | | Teeka akayini **(√)** mu ka box | | 🡪🡪🡪🡪🡪🡪🡪🡪🡪🡪 | |  | **EINFO1** |
| 7.3 | | **Motivation**  *O What would be some benefits to getting ART?*  *O What are your concerns about ART?*  *O What have been the not so good things that have happened or may happen by not getting ART?* | *O Bulungi ki obuli mukufuna eddagala erikendeeza obungi bwakawuka ka siriimu mu mubiri ( ARV)?*  *O Bweralikirivu ki bwolina ku ddagala lya ARV?*  *O Bintu ki ebitabadde birungi ebibaddewo oba ebiyinza okubaawo singa omuntu tafuna ddagala erikendeeza akawuka ka siriimu mumubiri?* | [Checkbox] | | Teeka akayini **(√)** mu ka box | | 🡪🡪🡪🡪🡪🡪🡪🡪🡪🡪 | |  | **EMOTI1** |
| 7.4 | | **Behavioral Skills**  O *How confident are you that you could get on ART if you wanted to?*  *O What are things that would make it difficult to get on ART even if you wanted to?* | O *Oli mukakafu kwenkanawa nti osobola okufuna eddagala erikendeeza obungi bwakawuka ka siriimu singa oba olyagadde?*  *O Bintu ki ebiyinza okuzibuwaza okufuna eddagala erikendeeza obungi bwakawuka ka siriimu wadde ng’oba olyetaaze?* | [Checkbox] | | Teeka akayini **(√)** mu ka box | | 🡪🡪🡪🡪🡪🡪🡪🡪🡪🡪 | |  | **EBEHA1** |
| 7.5 | | **Intentions**  *O Given how you feel right now, would you try to get started on ART?* | *O Okusinziira bwewewulira kati, wandigezezzaako okutandika okufuna eddagala erikendeeza obungi bwakawuka ka siriimu?* | Yes…………………. 1  No………………….. 0  Not sure……………. 77 | | Yee……………………...1  Nedda………………….. 0  Sikakasa……………….77 | | If 1, then skip to 7.7  Else, 🡪🡪🡪🡪🡪🡪 | | Bwekiba Yee, buuka ogende ku namba 7.7 | **EMOTI2** |
| 7.6 | | **Targets**  *O What would need to change for you to want to get started on ART?* | *O Biki ebyetaaga okukyuuka okukusobozesa okwagala okufuna eddagala erikendeeza obungi bw’ akawuka ka siriimu?* | [Checkbox] | | Teeka akayini **(√)** mu ka box | | 🡪🡪🡪🡪🡪🡪🡪🡪🡪🡪 | |  | **EMOTI3** |
| 7.7 | | *Some people are ready to start ART and some are not ready right now. Most people think that it is helpful to have information, no matter how ready they are. So if it is okay with you, I will share some additional information with you:*  O ART is free. **[I]**  O All persons with HIV in Kasensero are recommended to start ART. **[I]**  O To feel and look good, persons with HIV should take ART **[M]**  O If you have children, having them tested for HIV is a great step to making sure they are healthy. **[I,M]**  O If you need a referral to another health clinic while you are traveling, I can help you. **[I]** | Abantu abamu betegefu okutandika okumira eddagala erikendeeza obungi bw’ akawuka ka siriimu, ate abalala sibetegefu. Abantu abasinga balowooza nti kyamugaso okufuna okumanyisibwa, nebwebaba betegefu oba nedda. Bwekiba tekirina buzibu, nja kugabana naawe ebintu bino wammanga  O *sjabi bw’eddagala erikendeeza obungi bw’ akawuka ka siriimu mumubiri lyabwereere*. **[I]**  O *Abantu bonna abalina akawuka ka siriimu e Kasensero basaanidde okutandika okumira eddagala erikendeeza obungi bw’ akawuka ka siriimu*  **[I]**  OOkwewulira n’okufaanana obulungi, abantu abalina akawuka ka siriimu betaaga okumira eddagala erikendeeza obungi bw’ akawuka ka siriimu **[M]**  O Bwoba olina abaana, kikulu nnyo okubakebeza akawuka ka siriimu, okukakasa nti balamu . **[I,M]**  O Bwoba wetaaga okuweerezebwa ku kilinika endala, ng’obadde otambudde, nsobola okukuyamba **[I]** | [Checkbox] | | Teeka akayini **(√)** mu ka box | | 🡪🡪🡪🡪🡪🡪🡪🡪🡪🡪 | |  | **E1** |
| **F** | | **HIV-positive, On ART** | **Abalina akawuka ka siriimu, nga bamira eddagala erikendeeza obungi bw’ akawuka ka siriimu** |  | |  | |  | |  |  |
| 8.1 | | Please ask the client the following questions using the motivational interviewing skills you have learned. | Buuza ebibuuzo bino wammanga ng’okozesa obukugu obuzzaamu amaanyi mukubuuza ebibuuzo bw’oyize. |  | |  | | 🡪🡪🡪🡪🡪🡪🡪🡪🡪🡪 | |  |  |
| 8.2 | | **Information**  O *Can you tell me what you know about taking ART?*  O *What do you know about the availability of ART when you are not staying in Kasensero?* | *O Osobola okumbuulira by’omanyi ku kumira eddagala erikendeeza obungi bwakawuka ka siriimu mumubiri?* | [Checkbox] | | Teeka akayini **(√)** mu ka box | | 🡪🡪🡪🡪🡪🡪🡪🡪🡪🡪 | |  | **FINFO1** |
| 8.3 | | **Motivation**  *O What would be some benefits to taking your ART meds?*  *O What are your concerns about your ART?*  *O What are your concerns about taking your ART when you are traveling?*  *O What have been the not so good things that have happened or may happen by not taking ART?* | *O Birungi ki ebiri mukumira eddagala lyo erikendeeza obungi bwakawuka ka siriimu?*  *O Bweralikirivu ki bwolina ku ddagala lyo erikendeeza akawuka ka siriimu mu mubiri( ARV)?*  *O Bweralikirivu ki bwolina ku kumira eddagala lyo erikendeeza akawuka ka siriimu mu mubiri( ARV) ng’otambudde?*  *O Bintu ki ebitali birungi ebibaddewo oba ebiyinza okubaawo singa tomira ddagala lyo erikendeeza obungi bwakawuka ka siriimu mumubiri?* | [Checkbox] | | Teeka akayini **(√)** mu ka box | | 🡪🡪🡪🡪🡪🡪🡪🡪🡪🡪 | |  | **FMOTI1** |
| 8.4 | | **Behavioral Skills**  O *If your goal was to take your ART every day what would help you achieve that goal?*  O *Who or what could help to make sure you stay on ART even while you travel?*  *O And what could get in the way of achieving these goals?* | *O Singa ekigendererwakyo kyakumira ddagala lyo buli lunaku, kiki ekiyinza okukuyamba okutuukiriza ekigendererwa ekyo?*  *O Ani oba kiki ekiyinza okuyamba okulaba nti osigala ng’omira eddagala lyo erikendeeza obungi bwakawuka ka siriimu mumubiri, nebwoba otambudde?*  *O Kiki ekiyinza okukulemesa okutuukiriza ekigendererwa ekyo?* | [Checkbox] | | Teeka akayini **(√)** mu ka box | | 🡪🡪🡪🡪🡪🡪🡪🡪🡪🡪 | |  | **FBEHA1** |
| 8.5 | | **Intentions**  *O Given how you feel right now, will you try to take your ART every day?*  *O Given how you feel right now, do you plan on staying on ART when you are not in Kasensero?* | *O Okusinziira bwowulira kati,onogezaako okumira eddagala lyo erikendeeza obungi bwakawuka ka siriimu mumubirI buli lunaku?*  *O Okusinziira bwowulira kati , oteekateeka okusigala ng’omira eddagala erikendeeza obungi bwakawuka ka siriimu mumubiri, nebwoba toli mu Kasensero?* | Yes…………………. 1  No………………….. 0  Not sure……………. 77  Yes…………………. 1  No………………….. 0  Not sure……………. 77 | | Yee…………………. 1  Nedda………………….. 0  Sikakasa………………..77  Yee…………………. 1  Nedda………………….. 0  Sikakasa………………..77 | | If 1 to both, then skip to 8.7  Else, 🡪🡪🡪🡪🡪🡪 | | Bwekiba Yee kubibuuzo byombi,buuka ogende ku namba 8.7 | **FMOTI2**  **FMOTI2B** |
| 8.6 | | **Targets**  *O What would need to change for you to want to take your ART every day?*  *O What would need to change for you to want to stay on ART when you are not in Kasensero?* | *O Kiki ekyetaaga okukyuuka okukusobozesa okumira eddagala lyo erikendeeza obungi bwakawuka ka siriimu mumubiri buli lunaku?*  *O Kiki ekyetaaga okukyuuka okukusobozesa okumira eddagala lyo erikendeeza obungi bwakawuka ka siriimu mumubiri nga toli Kasensero?* | [Checkbox] | | Teeka akayini **(√)** mu ka box | | 🡪🡪🡪🡪🡪🡪🡪🡪🡪🡪 | |  | **FMOTI3** |
| 8.7 | | *If it is okay with you, I will share some additional information with you:*  O If you have children, having them tested for HIV is a great step to making sure they are healthy. **[I,M]**  O If you are on medicines, I can help you stay on them when you are traveling. **[B]**  O If you need a referral to another health clinic while you are traveling, I can help you. **[I]** | *Bwekiba tekirina buzibu, ngenda kugabana naawe okumanyisibwa okulala*  O Bwoba olina abaana, kikulu nnyo okubakebeza akawuka ka siriimu, okukakasa nti balamu . **[I,M]**  O bwoba oli ku ddagala, nsobola okukuyamba okulisigalako ng’otambula **[B]**  O Bwoba wetaaga okuweerezebwa ku kilinika endala ng’obadde otambudde, nsobola okukuyamba **[I]** | [Checkbox] | | Teeka akayini **(√)** mu ka box | | 🡪🡪🡪🡪🡪🡪🡪🡪🡪🡪 | |  |  |
|  | **G Pregnant+, HIV positive** | | | | | | | | | | |
| 9.1 | | Please ask the client the following questions using the motivational interviewing skills you have learned. | Buuza ebibuuzo bino wammanga ng’okozesa obukugu obuzzaamu amaanyi mukubuuza ebibuuzo bw’oyize. |  | |  | | 🡪🡪🡪🡪🡪🡪🡪🡪🡪🡪 | |  |  |
| 9.2 | | **Information**  O *Can you tell me what you know about taking ART when you are pregnant?* | *O Osobola okumbuulira kyomanyi kukumira eddagala*  *erikendeeza obungi bwakawuka ka siriimu mumubiri ng’oli lubuto?* | [Checkbox] | | Teeka akayini **(√)** mu ka box | | 🡪🡪🡪🡪🡪🡪🡪🡪🡪🡪 | |  | **GINFO1** |
| 9.3 | | **Motivation**  *O What would be some benefits to taking ART when pregnant?*  *O What are your concerns about taking ART when pregnant?* | *O Birungi ki ebiri mukumira eddagala erikendeeza obungi bwakawuka ka siriimu mumubiri, ng’oli lubuto?*  *O Bweralikirivu ki bwolina kukumira eddagala erikendeeza obungi bwakawuka ka siriimu mumubiri ng’oli lubuto?* | [Checkbox] | | Teeka akayini **(√)** mu ka box | | 🡪🡪🡪🡪🡪🡪🡪🡪🡪🡪 | |  | **GMOTI1** |
| 9.4 | | **Behavioral Skills**  O *Who or what would support you in taking ART during your pregnancy?*  *O What could get in the way of this goal?* | *O Ani oba kiki ekiyinza okukuymba okumira eddagala lyo erikendeeza obungi bwakawuka ka siriimu mumubiri ng’oli lubuto?*  *O Kiki ekiyinza okulemesa ekigendererwa kino?* | [Checkbox] | | Teeka akayini **(√)** mu ka box | | 🡪🡪🡪🡪🡪🡪🡪🡪🡪🡪 | |  | **GBEHA1** |
| 9.5 | | **Intentions**  *O Based on how you feel right now, do you plan to be on ART while pregnant?* | *O Okusinziira bwowulira kati, otegese okubeera ku ddagala*  *erikendeeza obungi bwakawuka ka siriimu mumubiri ng’oli lubuto?* | Yes…………………. 1  No………………….. 0  Not sure……………. 77 | | Yee…………………. 1  Nedda………………….. 0  Sikakasa………………..77 | | If 1, then skip to 9.7  Else, 🡪🡪🡪🡪🡪🡪 | | Bwekiba Yee, buuka ogende ku namba 9.7 | **GMOTI2** |
| 9.6 | | **Targets**  *O What would need to change for you to be on ART while pregnant?* | *O Kiki ekyetaaga okukyusa okukusobozesa okubeera ku ddagala* *erikendeeza obungi bwakawuka ka siriimu mumubiri ng’oli lubuto?* | [Checkbox] | | Teeka akayini **(√)** mu ka box | | 🡪🡪🡪🡪🡪🡪🡪🡪🡪🡪 | |  | **GMOTI3** |
| 9.7 | | *Some people are ready to get tested and some are not ready right now. Most people think that it is helpful to have information, no matter how ready they are. So if it is okay with you, I will share some additional information with you:*  O For pregnant women who are HIV-positive, getting and staying on ART is a great step to making sure they have a healthy baby. **[I,M]** | *Abantu abamu beetegefu okwekebeza akawuka ka siriimu, abamu sibetegefu kati.Abantu abasinga balowooza nti kyamugaso okufuna okumanyisibwa, nebwebaba betegefu oba nedda. N’olwekyo, bwekiba tekirina buzibu, nja kugabana naawe ebintu bino wammanga*  *O Abakyala abalina akawuka ka siriimu, okufuna n’okusigala nga bamira eddagala* *erikendeeza obungi bwakawuka ka siriimu, kintu kikulu nnyo okukakasa nti bazaala omwana omulamu*. **[I,M]** | [Checkbox] | | Teeka akayini **(√)** mu ka box | | 🡪🡪🡪🡪🡪🡪🡪🡪🡪🡪 | |  | **G1** |
| **H** | | **Risky Sex+, Male+** | **Empisa ez’obulabe mubyokwegatta (abaami)** |  | |  | |  | |  |  |
| 10.1 | | Please ask the client the following questions using the motivational interviewing skills you have learned. | Buuza ebibuuzo bino wammanga ng’okozesa obukugu obuzzaamu amaanyi mukubuuza ebibuuzo bw’oyize. |  | |  | | 🡪🡪🡪🡪🡪🡪🡪🡪🡪🡪 | |  |  |
| 10.2 | | **Information**  O *Can you tell me what you know about HIV transmission for men who do not always use a condom?* | O Osobola okumbuulira ky’omanyi kukusiiga/okusiigibwa kw’kawuka ka siriimu eri abasajja abatakozesa bupiira bwa kondomu buli lwebegatta mumukwano? | [Checkbox] | | Teeka akayini **(√)** mu ka box | | 🡪🡪🡪🡪🡪🡪🡪🡪🡪🡪 | |  | **HINFO1** |
| 10.3 | | **Motivation**  *O What would be some benefits to using a condom?*  *O What are your concerns about condoms?*  *O What have been the not so good things that have happened or may happen by not using condoms?* | *O Mugaso ki oguli mukukozesa obupiira mukwegatta muby’omukwano (kondomu)?*  *O Biki byeweralikirira ku bupiira (kondomu)?*  *O Biki ebitali birungi ebibaddewo oba ebiyinza okubaawo mubutakozesa bupiira?* | [Checkbox] | | Teeka akayini **(√)** mu ka box | | 🡪🡪🡪🡪🡪🡪🡪🡪🡪🡪 | |  | **HMOTI1** |
| 10.4 | | **Behavioral Skills**  O *If you decided to start using condoms, what would help you follow through on this goal?*  *O What could get in the way of using them?* | O *Singa osalawo okutandika okukozesa obupiira, kiki ekiyinza okukuyamba okutuukiriza ekigendererwakyo ekyo?*  *O Kiki ekiyinza okukulemesa okukozesa obupiira?* | [Checkbox] | | Teeka akayini **(√)** mu ka box | | 🡪🡪🡪🡪🡪🡪🡪🡪🡪🡪 | |  | **HBEHA1** |
| 10.5 | | **Intentions**  *O Given how you feel right now, do you plan on using condoms every time you have sex?* | *O Okusinziira bwowulira kati, otegese okukozesa obupiira bwa kondomu buli lwewegatta mu mukwano?* | Yes…………………. 1  No………………….. 0  Not sure……………. 77 | | Yee…………………. 1  Nedda………………….. 0  Sikakasa……………….77 | | If 1, then skip to 10.7  Else, 🡪🡪🡪🡪🡪🡪🡪 | | Bwekiba Yee, buuka ogende ku namba 10.7 | **HMOTI2** |
| 10.6 | | **Targets**  *O What would need to change for you to want to use condoms more?* | *O Kiki ekyetaaga okukyuuka okukusobozesa okwagala okukozesa obupiira?* | [Checkbox] | | Teeka akayini **(√)** mu ka box | | 🡪🡪🡪🡪🡪🡪🡪🡪🡪🡪 | |  | **HMOTI3** |
| 10.7 | | *Some people are ready to use condoms and some are not ready right now. Most people think that it is helpful to have information, no matter how ready they are. So if it is okay with you, I will share some additional information with you:*  O Condoms are available for free through myself and the RHSP clinic. **[I, B]**  O Do you know how to use a condom? **[B]** | *Abantu abamu betegefu okukozesa obupiira bwa kondomu, abamu sibetegefu.*  *Abantu abasinga balowooza nti kyamugaso okufuna okumanyisibwa, nebwebaba betegefu oba nedda. N’olwekyo, bwekiba tekirina buzibu, nja kugabana naawe ebintu bino wammanga*  O Obupiira bwa kondomu weebuli kubwerere okuyita gyendi oba ku kilinika ya RHSP **[I, B]**  O Omanyi okukozesa akapiira ka kondomu? **[B]** | [Checkbox] | | Teeka akayini **(√)** mu ka box | | 🡪🡪🡪🡪🡪🡪🡪🡪🡪🡪 | |  | **H1** |
| **I** | | **Risky Sex+, Female+** | **Empisa ez’obulabe mubyokwegatta (abakyala)** |  | |  | |  | |  |  |
| 11.1 | | Please ask the client the following questions using the motivational interviewing skills you have learned. | Buuza ebibuuzo bino wammanga ng’okozesa obukugu obuzzaamu amaanyi mukubuuza ebibuuzo bw’oyize. |  | |  | | 🡪🡪🡪🡪🡪🡪🡪🡪🡪🡪 | |  |  |
| 11.2 | | **Information**  *O Can you tell me what you know about male condoms and HIV transmission?* | *O Osobola okumbuulira by’omanyi ku bupiira (kondomu) bwa basajja ne nsasanya y’akawuka ka siriimu?* | [Checkbox] | | Teeka akayini **(√)** mu ka box | | 🡪🡪🡪🡪🡪🡪🡪🡪🡪🡪 | |  | **IINFO1** |
| 11.3 | | **Motivation**  *O What would be some benefits to you having your partner use a condom?*  *O What are your concerns about being able to use condoms with your partner or partners?*  *O What have been the not so good things that have happened or may happen by not having condoms used by your partner or partners?*  *O What are your concerns about discussing condom use with your partner?* | *O Birungi ki ebiri mukukozesa obupiira bwa kondomu n’omwagalwa wo?*  *O Biki ebikweralikiriza ku kusobola okukozesa obupiira bwa kondomu n’omwagalwa oba abagalwa bo?*  *O Bintu ki ebitali birungi ebibaddewo oba ebiyinza okubaawo ng’omwagalwa oba abagalwa bo tebakozesezza bupiira bwa kondomu?*  *O Biki ebikweralikiriza ku kukubaganya ebirowoozo kukukozesa obupiira bwa kondomu n’omwagalwa oba abagalwa bo?* | [Checkbox] | | Teeka akayini **(√)** mu ka box | | 🡪🡪🡪🡪🡪🡪🡪🡪🡪🡪 | |  | **IMOTI1** |
| 11.4 | | **Behavioral Skills**  O *How confident are you that you could discuss condom use with your partner?*  *O What would make discussing condom use something difficult to do?* | O Wekakasa kyenkanawa nti osobola okukubaganya ebirowooza ku kyokukozesa obupiira bwa kondomu,n’omuntu gwewegatta naye mubyomukwano?  O Kiki ekiyinza okuzibuwaza okukubaganya ebirowoozo ku kyokukozesa obupiira bwa kondomu? | [Checkbox] | | Teeka akayini **(√)** mu ka box | | 🡪🡪🡪🡪🡪🡪🡪🡪🡪🡪 | |  | **IBEHA1** |
| 11.5 | | **Intentions**  *O Given how you feel right now, do you plan on discussing condom use with your partner?* | O Okusinziira bwowulira kati, oteekateeka okukubaganya ebirowoozo ku kyokukozesa obupiira bwa kondomu,n’omuntu gwewegatta naye mubyomukwano? | Yes…………………. 1  No………………….. 0  Not sure……………. 77 | | Yee…………………. 1  Nedda………………….. 0  Sikakasa……………….77 | | If 1, then skip to 11.7  Else, 🡪🡪🡪🡪🡪 | | Bwekiba Yee, buuka ogende ku namba 11.7 | **IMOTI2** |
| 11.6 | | **Targets**  *O What would need to change for you to want to use condoms more?* | *O Kiki ekyetaaga okukyuuka okukusobozesa okwagala okwongera okukozesa obupiira bwa kondomu?* | [Checkbox] | | Teeka akayini **(√)** mu ka box | | 🡪🡪🡪🡪🡪🡪🡪🡪🡪🡪 | |  | **IMOTI3** |
| 11.7 | | *Some people are ready to use condoms and some are not ready right now. Most people think that it is helpful to have information, no matter how ready they are. So if it is okay with you, I will share some additional information with you:*  O Condoms are available for free through myself and the RHSP clinic. **[I, B]**  O Do you know how to use a condom? **[B]** | *Abantu abamu betegefu okukozesa obupiira bwa kondomu, abamu sibetegefu.*  *Abantu abasinga balowooza nti kyamugaso okufuna okumanyisibwa, nebwebaba betegefu oba nedda. N’olwekyo, bwekiba tekirina buzibu, nja kugabana naawe ebintu bino wammanga*  O Obupiira bwa kondomu weebuli kubwerere okuyita gyendi oba ku kilinika ya RHSP **[I, B]**  O Omanyi okukozesa akapiira ka kondomu? **[B]** | [Checkbox] | | Teeka akayini **(√)** mu ka box | | 🡪🡪🡪🡪🡪🡪🡪🡪🡪🡪 | |  | **I1** |
| **J** | | **Mobile Phone** |  |  | |  | |  | |  |  |
| 12.1 | | *I will be following up with you over the next several months to continue these sessions. I may call or text you on your mobile phone number to arrange an appointment. Please let me know if your number changes.* | Nga bwennagambye olubereberye, nja kukugoberera mu myezi egijja okwongera okukubudabuda.  Nyinza okukubirra essimu oba okukuwereza ka message tukole entegeka.  Mubiseera ebyomumaaso, ennamba y’essimu yo wekyuka nga, ontegezza. | [Checkbox] | | Teeka akayini **(√)** mu ka box | | 🡪🡪🡪🡪🡪🡪🡪🡪🡪🡪 | |  | CONTACT |
| **K** | | **Wrap-Up** |  |  | |  | |  | |  |  |
| 13.1 | | O *Do you have any remaining questions or concerns about HIV and staying healthy?*  *O Thank you very much for allowing me to support you.* | O olinayo ebibuuzo ebirala oba ekikwata ku kawuka ka siriimu oba okusigala ng’oli mulamu?  *O Webale nnyo okunzikiriza okukuyamba* | [Checkbox] | | Teeka akayini **(√)** mu ka box | | 🡪🡪🡪🡪🡪🡪🡪🡪🡪🡪 | |  | WU1  WU2 |
| 13.2 | | Locate the Log Book form for this participant and enter the following information:  *O* Comments and/or Next Appointment |  | [Checkbox] | | Teeka akayini **(√)** mu ka box | | 🡪🡪🡪🡪🡪🡪🡪🡪🡪🡪 | |  |  |
| 13.2 | | Counseling complete.  Additional Participants? | Okubudabuda kukomye wano.  Waliyo abokubudabuda abalala? | Yes…………………. 1  No………………….. 0 | | Yee…………………. 1  Nedda…………………. 0 | | If 1, then back to 1.8  If 0, then continue to 13.3 | | Bwekiba Yee, ddayo ku namba 1.8  Bweba Nedda, genda ku 13.3 |  |
| 13.3 | | [Close application] | Ggalawo | [Buttons] | |  | | End of Algorithm. | | Koma awo |  |
